# The *Listeria* Small RNA Rli27 Regulates a Cell Wall Protein inside Eukaryotic Cells by Targeting a Long 5′-UTR Variant

**DOI:** 10.1371/journal.pgen.1004765

**Published:** 2014-10-30

**Authors:** Juan J. Quereda, Álvaro D. Ortega, M. Graciela Pucciarelli, Francisco García-del Portillo

**Affiliations:** 1 Centro Nacional de Biotecnología-Consejo Superior de Investigaciones Científicas (CNB-CSIC), Madrid, Spain; 2 Departamento de Biología Molecular, Universidad Autónoma de Madrid, Centro de Biología Molecular ‘Severo Ochoa’ (CBMSO-CSIC), Madrid, Spain; The University of Texas Health Science Center at Houston, United States of America

## Abstract

*Listeria monocytogenes* is a bacterial pathogen whose genome encodes many cell wall proteins that bind covalently to peptidoglycan. Some members of this protein family have a key role in virulence, and recent studies show that some of these, such as Lmo0514, are upregulated in bacteria that colonize eukaryotic cells. The regulatory mechanisms that lead to these changes in cell wall proteins remain poorly characterized. Here we studied the regulation responsible for increased Lmo0514 protein levels in intracellular bacteria. The amount of this protein increased markedly in intracellular bacteria (>200-fold), which greatly exceeded the increase in *lmo0514* transcript levels (∼6-fold). Rapid amplification of 5′-cDNA ends (RACE) assays identified two *lmo0514* transcripts with 5′-untranslated regions (5′-UTR) of 28 and 234 nucleotides. The transcript containing the long 5′-UTR is upregulated by intracellular bacteria. The 234-nucleotide 5′-UTR is also the target of a small RNA (sRNA) denoted Rli27, which we identified by bioinformatics analysis as having extensive base pairing potential with the long 5′-UTR. The interaction is predicted to increase accessibility of the Shine-Dalgarno sequence occluded in the long 5′-UTR and thus to promote Lmo0514 protein production inside the eukaryotic cell. Real-time quantitative PCR showed that Rli27 is upregulated in intracellular bacteria. *In vivo* experiments indicated a decrease in Lmo0514 protein levels in intracellular bacteria that lacked Rli27. Wild-type Lmo0514 levels were restored by expressing the wild-type Rli27 molecule but not a mutated version unable to interact with the *lmo0514* long 5′-UTR. These findings emphasize how 5′-UTR length affects regulation by defined sRNA. In addition, they demonstrate how alterations in the relative abundance of two transcripts with distinct 5′-UTR confine the action of an sRNA for a specific target to bacteria that occupy the intracellular eukaryotic niche.

## Author Summary

*Listeria monocytogenes* has evolved to adapt to numerous environments, including the intracellular niche of eukaryotic cells. Small RNAs (sRNA) play important regulatory roles in changing environments, and are thus predicted to modulate *L. monocytogenes* adaption to the intracellular lifestyle. This study shows how the regulatory activity of an sRNA on a defined target is restricted to bacteria in the intracellular infection phase. This regulation relies on a long (234-nucleotide) 5′-UTR that bears the sRNA-binding site present in a transcript variant that is upregulated by intracellular *L. monocytogenes*. The concomitant increase in both the target transcript containing the long 5′-UTR and the sRNA, which is postulated to facilitate opening of the Shine-Dalgarno site, culminates in markedly higher protein levels in intracellular bacteria. The limited amounts of both the target and the regulator in extracellular bacteria ensure that production of this bacterial protein is confined mainly to the host rather than the non-host environment.

## Introduction

*Listeria monocytogenes* is a facultative intracellular food-borne bacterium responsible for serious clinical manifestations including febrile gastroenteritis, meningitis, encephalitis and maternofetal infections in humans and livestock, with an estimated fatality rate of 20–30% of infected individuals [1]–[3]. Following ingestion, *L. monocytogenes* is able to cross the intestinal, blood-brain and placental barriers. The bacterium expresses a number of virulence factors that promote entry into phagocytic and non-phagocytic eukaryotic cells, intracellular survival and proliferation, and spreading to adjacent cells [4].

Genome studies show that all *Listeria* species sequenced to date have more than 40 genes that encode predicted surface proteins bearing an LPXTG sorting motif [5]. This motif is recognized by sortase enzymes, which anchor these proteins covalently to the cell wall. In pathogenic *Listeria*, some of these LPXTG proteins direct essential steps throughout the infection process, including bacterial adhesion and uptake by the host cell [6]–[9]. Proteomic analyses indicated that levels of many of these LPXTG surface proteins change on adaptation to different environments. The *Listeria* cell wall subproteome thus changes substantially in actively growing and resting bacteria. Mutants that lack sortase SrtA and SrtB activity show impaired LPXTG protein anchoring to the peptidoglycan [10] as well as differences in the relative levels of certain LPXTG proteins [11]. Recent studies also showed major changes in the cell wall proteome when *L. monocytogenes* proliferate inside epithelial cells [12]. Upregulation of defined LPXTG proteins has been observed in intracellular bacteria, including Internalin-A and Lmo0514 [12]. The mechanisms that regulate the coordinated production of such a large number of LPXTG proteins nonetheless remain largely unknown.

Bacterial small RNAs (sRNA) are a class of bacterial gene expression regulators important in many physiological processes, including virulence and cell envelope homeostasis [13], [14]. sRNA coordinate target gene expression in response to environmental changes and have regulatory functions that affect protein activity and mRNA stability/translation in many microorganisms, including bacterial pathogens [15], [16]. More than 100 sRNA have been identified for *L. monocytogenes* by the use of tiling arrays, global RNA sequencing (RNA-Seq) and bioinformatics methods [14], [17], [18]; more than 30 of these have been validated by northern blot, but their biological function and mechanisms of action are so far unknown [19]. There is little information on the regulation of sRNA expression in *L. monocytogenes*. Some reports implicate the alternative sigma factor SigB in regulating expression of the sRNA SbrA (Rli11) and SbrE (Rli47) [17], [20], [21]. In addition, 22 sRNA genes are preceded by putative sigma A boxes in the *L. monocytogenes* genome [14]. Recent studies also show that the sRNAs Rli31, Rli33-1, Rli38 and Rli50 modulate virulence in *L. monocytogenes* [14], [17]. Despite these studies, there is no model that describes how sRNA expression in *L. monocytogenes* responds to infection of eukaryotic cells. With the exception of LhrA, which controls expression of the chitinase ChiA post-transcriptionally [22], and of the multicopy sRNA LhrC, which modulates LapB adhesin expression [23], the identity of the functions targeted by *L. monocytogenes* sRNA inside or outside eukaryotic cells, remains unknown.

Here we studied the regulatory mechanism responsible for the increase in the LPXTG protein Lmo0514 in the cell wall of intracellular bacteria [12]. Our data demonstrate an sRNA that is a key regulatory element in modulating levels of this cell wall surface protein during intracellular infection. This response to the eukaryotic niche is directed by the activity of two promoters in the target gene that generate transcripts with 5′-untranslated regions (5′-UTR) of distinct length. The relative abundance of these two transcripts differs in extra- and intracellular bacteria. Only the ‘long’ version, enriched in intracellular bacteria, bears the sRNA binding site. This mechanism confines the regulation of *lmo0514* by this sRNA to the intracellular eukaryotic niche.

## Results

### The *L. monocytogenes* gene that encodes the LPXTG surface protein Lmo0514 is expressed as two variants with distinct 5′-UTR

**Figure 1:**
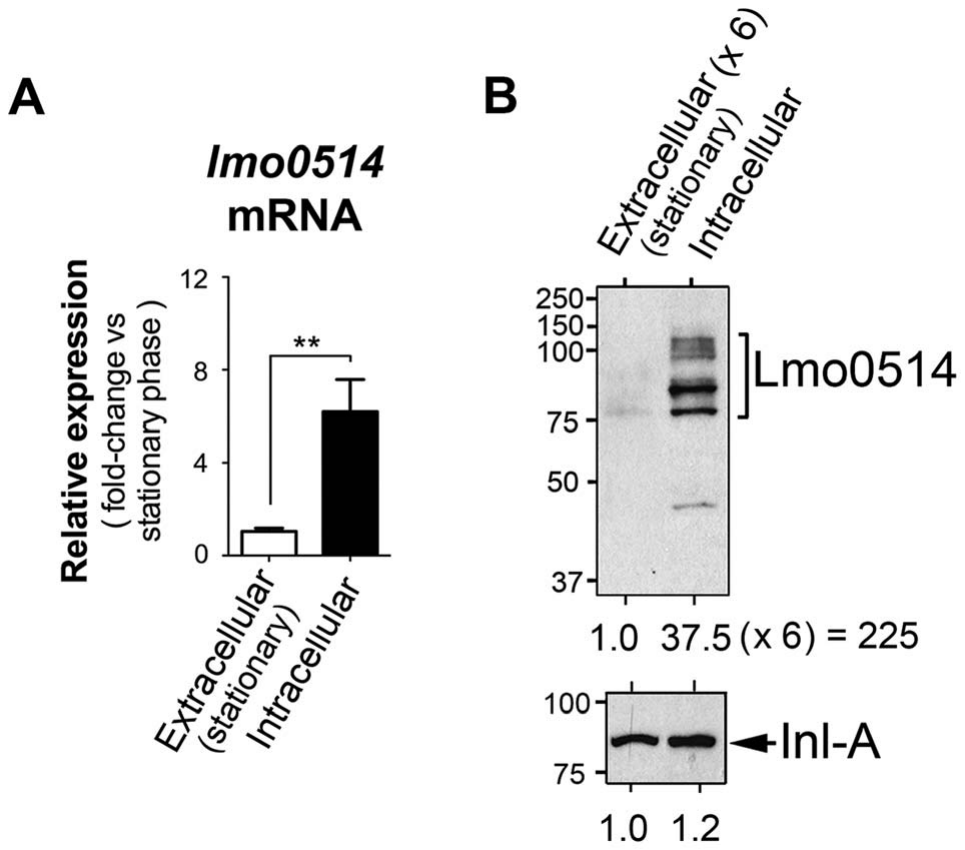
Regulation of the *L. monocytogenes* gene that encodes the LPXTG surface protein Lmo0514 in intracellular bacteria located inside eukaryotic cells. Total RNA and cell wall protein extracts were prepared from *L. monocytogenes* wild-type strain EGD-e grown in BHI medium to stationary phase (extracellular) and from bacteria collected in JEG-3 epithelial cells at 6 h post-infection (intracellular). (A) *lmo0514* transcript levels monitored by real-time qPCR using primers Lmo0514-F and Lmo0514-R, which map within the *lmo0514* coding region (Table S2). (B) Lmo0514 protein levels detected by Western blot. Levels of another LPXTG surface protein, Internalin-A (Inl-A), are shown for comparison. Densitometry analysis of bands is shown as numbers relative to the band detected in wild-type bacteria. Cell wall extracts of extracellular bacteria are concentrated 6-fold relative to those of intracellular bacteria. In intracellular bacteria, note the marked increase in relative levels of Lmo0514 protein (37.5×6 = 225), which contrasts with the ∼6-fold increase in *lmo0514* mRNA.

Lmo0514, a *L. monocytogenes* LPXTG surface protein of unknown function, is encoded by a gene upregulated by bacteria located within macrophages [24]. Lmo0514 is also more abundant in the cell wall of bacteria that proliferate inside epithelial cells than in bacteria growing in laboratory media [12]. To study the basis of this regulation, we compared *lmo0514* expression in extra- and intracellular bacteria. Real-time quantitative PCR (qPCR) assays showed enhanced *lmo0514* mRNA expression (∼6-fold) in intracellular bacteria after infection of JEG-3 human epithelial cells (Fig. 1A). Consistent with our previous work [12], the Lmo0514 protein was detected mainly in the cell wall of intracellular bacteria, with very low levels in extracellular bacteria (Fig. 1B). Changes in relative levels of Lmo0514 protein were estimated to be>200-fold (Fig. 1B), much higher than those for *lmo0514* mRNA (∼6-fold). This lack of correlation between induction of *lmo0514* transcript and protein levels in intracellular bacteria led us to hypothesize that post-transcriptional regulatory mechanisms act on this gene.

**Figure 2:**
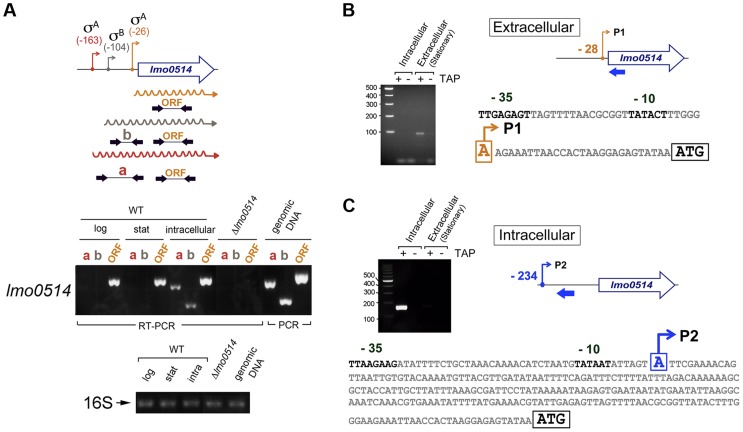
*lmo0514* is expressed differentially from two distinct transcriptional start sites in extra- and intracellular *L. monocytogenes*. (A) Position of the three transcriptional start sites (TSS) predicted *in silico* for *lmo0514* by Loh *et al.* [25]. Primers used to amplify the *lmo0514* coding sequence are indicated (ORF, primers Lmo0514-F, Lmo0514-R, see Table S2), as well as two fragments of the 5′-UTR of different lengths, amplicon “a” (254 nt), obtained with primers UTR-B and UTR-1R (Table S2) and amplicon “b” (134 nt), obtained with primers UTR-A and UTR-1R (Table S2). Reverse transcriptase-PCR assays showing upregulation in intracellular bacteria of an *lmo0514* transcript isoform with a long 5′-UTR. 16S rRNA was monitored as loading control. RNA was obtained from extracellular bacteria grown in BHI medium to exponential logarithmic phase (log), stationary phase (stat), and from intracellular bacteria. (B) 5′-RACE assay showing a TSS at position −28 relative to the ATG site in extracellular bacteria. Colored bar indicates the position of the primer lmo0514-PE-1rv (Table S2) used for this reaction. (C) 5′-RACE assay showing the production by intracellular bacteria of an *lmo0514* transcript with a long 5′-UTR derived from a TSS at position −234 relative to the ATG site. Colored bar indicates the position of the primer lmo0514-PE-6rv (Table S2) used for this reaction. TAP, tobacco acid pyrophosphatase.

**Figure 3:**
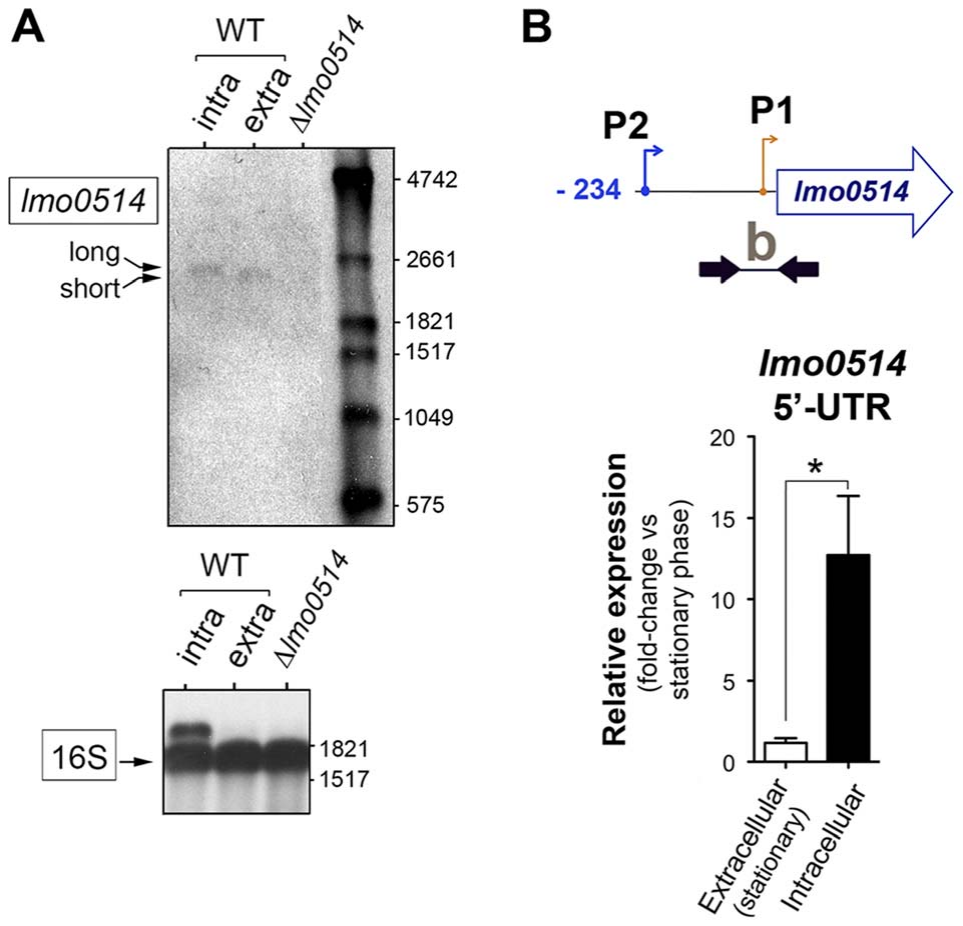
Northern blot and real-time quantitative PCR (qPCR) assays confirm the predominance of *lmo0514* transcripts of different lengths in extra- and intracellular *L. monocytogenes*. (A) Northern blot assays showing the short and long *lmo0514* transcript isoforms in RNA isolated from extra- and intracellular bacteria, respectively. Transcript size is compatible with cotranscription of *lmo0514* with downstream gene *lmo0515* (Fig. S1). Relative 16S rRNA levels are shown for comparison. (B) Relative *lmo0514* transcript levels detected by qPCR in extra- and intracellular bacteria, with Utr0514_qPCR_F and Utr0514_qPCR_R primers (Table S2) specific for the 5′-UTR. Data derived from a minimum of three independent experiments. *, *P*≤0.05, Student's *t*-test.

To evaluate this possibility, we sought *lmo0514* gene expression control mechanisms that operate specifically in intracellular bacteria. Previous *in silico* predictions by Loh et al. [25] indicated that *lmo0514* could be expressed from three promoters at positions −26, −104 and −163. Two of these, −26 and −163, were assigned as tentatively regulated by sigma A (σ^A^) and the third, at position −104, as controlled by sigma B (σ^B^) [25] (Fig. 2A). The activity of these putative promoters and the presence of the different transcripts were analyzed by RT-PCR on RNA isolated from *L. monocytogenes* grown extracellularly and from intracellular bacteria that colonized JEG-3 epithelial cells. *lmo0514* transcripts with a long 5′-UTR were detected specifically in intracellular bacteria (Fig. 2A). To confirm these findings, rapid amplification of 5′-cDNA ends (5′-RACE) assays were used to map transcriptional start sites (TSS) of *lmo0514* in bacteria grown extracellularly and in bacteria isolated from eukaryotic cells. These 5′-RACE assays revealed two distinct TSS at positions −28 and −234 (Fig. 2B, C), and also confirmed expression of the long *lmo0514* transcript by intracellular bacteria (Fig. 2C). Putative promoters for these TSS, which we termed P1 and P2, both bear bona fide −10 TATA boxes (Fig. 2B, C). The existence of two *lmo0514* transcripts of different length was verified by northern blot (Fig. 3A), with sizes compatible with cotranscription of *lmo0514* with the downstream gene *lmo0515*, which encodes a universal stress protein [26]. *lmo0514*-*lm0515* cotranscription was verified by RT-PCR (Fig. S1). qRT-PCR assays confirmed that expression of the *lmo0514* transcript variant with the long 234-nucleotide (nt) 5′-UTR was upregulated by ∼12-fold in intracellular bacteria (Fig. 3B). These findings suggested that the specific induction of this mRNA variant with a longer 5′-UTR in intracellular bacteria accounts for or contributes to the 6-fold increase in total *lmo0514* mRNA (Fig. 1A). These data supported a model in which intracellular bacteria specifically upregulate expression from the P2 promoter, resulting in an *lmo0514* transcript with a long 5′-UTR. This assumption takes into account the different ratios between the two *lmo0514* transcripts when *L. monocytogenes* colonizes the eukaryotic cell.

### The *lmo0514* long 5′-UTR variant has a binding site for Rli27, an sRNA induced in intracellular bacteria

The increased length of the *lmo0514* transcript variant that is upregulated in intracellular bacteria prompted us to test whether the distinctive 234-nt 5′-UTR is a target region for sRNA-mediated post-transcriptional regulation. We used *in silico* analysis to search for putative non-coding RNAs in the *L. monocytogenes* reference strain EGDe [27] that could bind to this *lmo0514* long 5′-UTR. The targetRNA program (http://cs.wellesley.edu/~btjaden/TargetRNA2/) [28] gave a high score to a pairing between defined stretches of the *lmo0514* 234-nt 5′-UTR and a sequence in the *lmo0411-lmo0412* intergenic region. A gene in this region encodes an sRNA termed Rli27 that is upregulated by *L. monocytogenes* in the intestine of infected mice and in human blood, as shown by transcriptomics [17]; RNA-seq corroborated the expression of this sRNA [18]. Although Rli27 was identified as an sRNA induced in infection conditions [17], no further characterization of its function or targets was reported.

**Figure 4:**
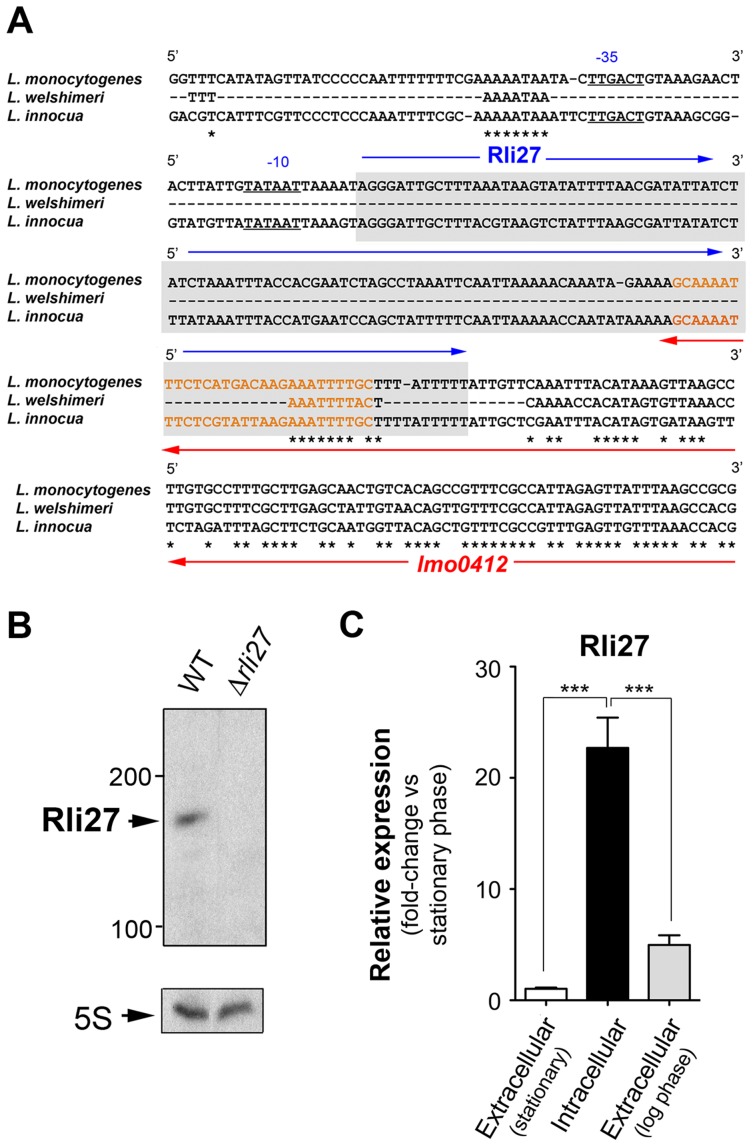
Rli27 is a bona fide *L. monocytogenes* sRNA induced by intracellular bacteria. (A) Sequence alignment of the *rli27* genomic region from *L. monocytogenes*, *L. welshimeri* and *L. innocua*. The −35 and −10 predicted sites for the *rli27* promoter and the *rli27* itself (grey background) are highlighted. Nucleotide sequence in orange corresponds to the predicted terminator shared by *rli27* and *lmo0412*. Note that *rli27* is absent in *L. welshimeri*. (B) Northern blot assay performed with total RNAs isolated from bacteria grown in BHI medium to stationary phase. *L. monocytogenes* strains used included EGDe (WT) and the Δ*rli27* mutant. 5S rRNA was used as loading control. (C) Real-time qPCR showing upregulation of Rli27 expression in intracellular bacteria. Bacteria were grown in BHI medium to exponential (log) or stationary phase, or collected from epithelial cells. Data derive from a minimum of three independent experiments. ***, *P*≤0.001, Student's *t*-test.

Genomic comparisons of pathogenic and non-pathogenic species are usually carried out to identify virulence genes, including sRNAs [18], [29]. We analyzed the genomic region of *L. monocytogenes* containing *rli27* and those of the non-pathogenic species *L. innocua* and *L. welshimeri*. In *L. monocytogenes*, *rli27* is flanked by *lmo0411* and *lmo0412*, two genes that map in the opposite DNA strand (Fig. S2), whereas in the *L. welshimeri* genome, the same intergenic region has a small ORF (*lwe0373*) that codes for a predicted protein of unknown function (Fig. S2). We nonetheless found that Rli27 is highly conserved in *L. innocua* (82% identity, Fig. 4A), in contrast with a previous report [17]. The extremely variable *rli27* genomic region might thus have been shaped by gain and/or loss of genes during *Listeria* speciation. Apart from *Listeria* species, BLAST searches did not identify *rli27* orthologs in other bacterial species. Rli27, identified as a 131-nt sRNA [14], [18], is not predicted to encode any protein using the Small Open Reading Frame (ORF) tool in the ORF finder program (http://www.bioinformatics.org/sms2/orf_find.html).

Although the existence of Rli27 sRNA was inferred based on its detection by genomic and transcriptomic approaches, it has not yet been formally demonstrated. The presence of *rli27* and its flanking genes in different strands ruled out the possibility that its detection by tiling arrays and RNA-seq analyses was due to untranslated regions of neighbor genes. *rli27* has its own predicted transcription start site and Rho-independent terminator sequence (Fig. 4A), and the respective promoter regions in *L. monocytogenes* and *L. innocua* showed no significant divergence (Fig. 4A). Northern blot assays using total RNA isolated from *L. monocytogenes* wild-type EGD-e and an isogenic Δ*rli27* mutant strain demonstrated a small transcript consistent with the ascertained size of Rli27 (∼130 nt) (Fig. 4B). Real-time qPCR showed that Rli27 expression is induced (∼20-fold) in intracellular bacteria when compared with extracellular bacteria grown in rich medium to logarithmic or stationary phases (Fig. 4C). These findings indicate that Rli27 is a bona fide sRNA that is upregulated by *L. monocytogenes* inside eukaryotic cells.

### Rli27 interacts physically with the 5′-UTR specific to the *lmo0514* long transcript

**Figure 5:**
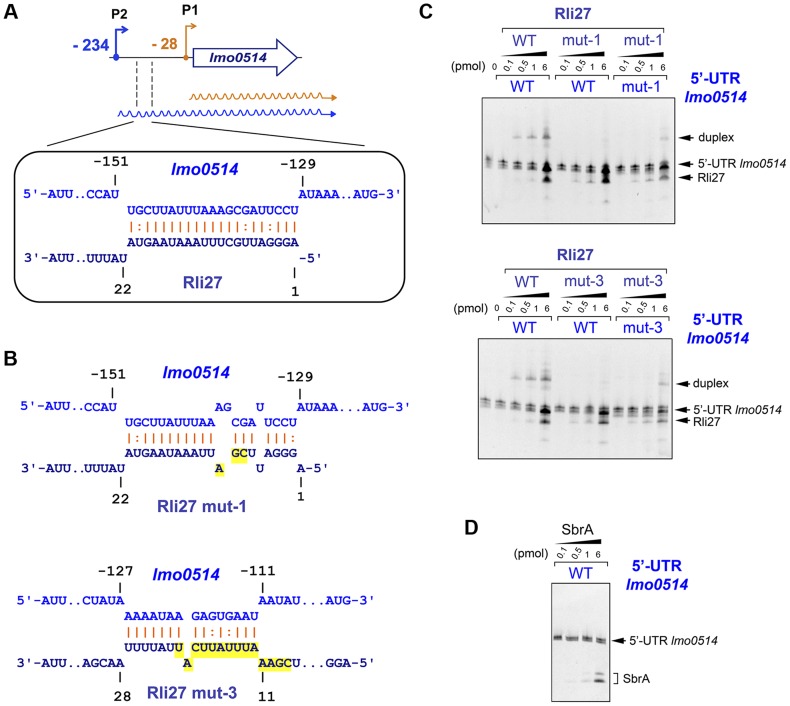
Rli27 interacts *in vitro* with the *lmo0514* long 5′-UTR. (A) Scheme of the major interaction region between Rli27 and the *lmo0514* 5′-UTR predicted with the targetRNA program (http://cs.wellesley.edu/~btjaden/TargetRNA2/). The complete set of putative interaction sites is shown in Fig. S3. (B) Effect of Rli27-mut1 and Rli27-mut3 mutations on the predicted Rli27-5′-UTR-*lmo0514* interaction. Changes are highlighted in yellow. Compensatory mutations designed in 5′-UTR molecules synthesized *in vitro* are also shown in Fig. S3. (C) EMSA assays showing formation of a 5′-UTR-*lmo0514*/Rli27 duplex with slow migration in the gel. This duplex is not formed after co-incubation of the 5′-UTR molecule with the Rli27-mut1 or Rli27-mut3 variants, and is partially restored by compensatory mutations in the *lmo0514* 5′-UTR. (D) Control EMSA showing no duplex formation after incubation of the *lmo0514* 5′-UTR with an unrelated sRNA, SbrA.

Rli27 interaction with the *lmo0514* 5′-UTR extends to several regions, although it shows a major predicted pairing region involving Rli27 nucleotides 1 to 21 (Fig. 5A, Fig. S3). We used electrophoretic mobility shift assays (EMSA) to assess the validity of this prediction. We generated *in vitro* wild-type versions of Rli27 and 5′-UTR-*lmo0514*, together with variants of both RNA molecules bearing mutations in 3 nt (mut-1) or 14 nt (mut-3) important for pairing (Fig. 5B). Incubation of Rli27 and 5′-UTR-*lmo0514* wild-type molecules resulted in a duplex with low electrophoretic mobility (Fig. 5C). Conversely, combination of wild-type 5′-UTR-*lmo0514* with mutated Rli27 (either mut-1 or mut-3 variants), reduced duplex formation (Fig. 5C). Duplex formation was partially restored by combining mutations in Rli27 with compensatory mutations in 5′-UTR-*lmo0514* (Fig. 5C). Specificity of the Rli27-5′-UTR-*lmo0514* interaction was confirmed by lack of duplex formation after incubation of the 5′-UTR-*lmo0514* wild-type molecule with SbrA, an unrelated sRNA (Fig. 5D).

### Rli27 interaction with the *lmo0514* long 5′-UTR is necessary to increase Lmo0514 protein levels in intracellular bacteria

**Figure 6:**
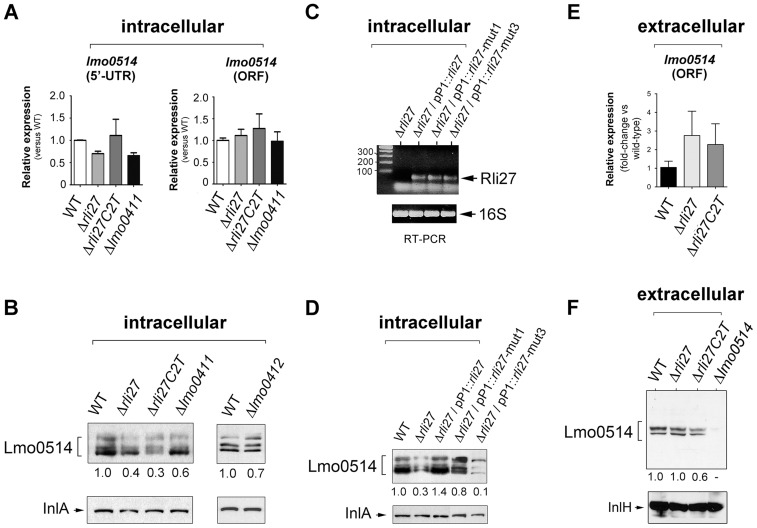
Rli27 is necessary for upregulation of Lmo0514 protein levels in intracellular bacteria by a mechanism that involves interaction with the *lmo0514* long 5′-UTR isoform. (A) Real-time qPCR assays showing no changes in relative levels of the *lmo0514* ORF (fragment amplified with Lmo0514-F and Lmo0514-R primers) or the isoform bearing the long 5′-UTR (fragment amplified with UTR-B and UTR-1R primers) in intracellular bacteria that lack Rli27 or are defective for the *rli27*-flanking *lmo0411* gene. (B) Decrease in levels of Lmo0514 protein produced by intracellular bacteria, caused by absence of Rli27. No such effect was seen in *lmo0411*- or *lmo0412*-defective strains. Levels of InlA, another cell wall-bound LPXTG protein, were monitored as loading control. (C) RT-PCR assays showing expression of Rli27 and mutated versions Rli27-mut1 and Rli27-mut3 produced in trans from the pP1 plasmid. (D) Western blot assays showing the effect of the mut3 mutation in Rli27, which impedes upregulation of the Lmo0514 protein in intracellular bacteria. InlA levels were monitored as loading control. (E) Real-time qPCR showing that lack of Rli27 does not affect *lmo0514* transcript stability in extracellular bacteria grown in BHI medium to stationary phase. Primers correspond to those that map in the coding region (Lmo0514-F and Lmo0514-R). Data are derived from a minimum of three independent experiments. (F) Western blot showing that Rli27 is not necessary for Lmo0514 protein production by extracellular bacteria. Due to the small amount of Lmo0514 protein produced by extracellular bacteria (see Fig. 1B), the anti-Lmo0514 antibody-treated membrane was overexposed. Levels of InlH, another cell wall-bound LPXTG protein, were monitored as loading control. Densitometry values are indicated in Western blots beneath panels B, D, and F.

To determine the biological relevance of the 5′-UTR-*lmo0514*-Rli27 interaction *in vivo*, we analyzed the specific contribution of Rli27 binding to Lmo0514 protein upregulation in bacteria that infect eukaryotic cells. We generated a Δ*rli27* strain and a second isogenic mutant, Δ*rli27C2T*, which bears an artificial strong terminator between the remaining *rli27* sequences. This mutant was intended to avoid polar effects on the flanking genes *lmo0411* and *lmo0412* (Fig. S4); we also included mutants in these flanking genes, Δ*lmo0411* and Δ*lmo0412* [30]. In addition, we designed a qPCR assay specific for the *lmo0514* long 5′-UTR for comparison to the *lmo0514* coding region. There were no notable differences among strains in the relative levels of the long 5′-UTR region or the *lmo0514* ORF (Fig. 6A). In contrast, Lmo0514 protein levels were ∼2.5- to 3-fold lower in the cell wall of the two Rli27-lacking mutant strains isolated from the eukaryotic cell (Fig. 6B). This phenotype was complemented by overproduction of wild-type or mut1 versions of Rli27 from a plasmid (Fig. 6C, D). In contrast, when we tested mut3, the Rli27 mutant bearing 14 nt changes in the major region predicted to interact with the *lmo0514* 5′-UTR (Fig. 5A), it did not restore Lmo0514 protein levels in intracellular bacteria (Fig. 6D). Wild-type, mut1 and mut3 Rli27 versions were all produced by the plasmid at similar levels (Fig. 6C). These data showed that Rli27 interaction with the *lmo0514* long 5′-UTR was essential for induction of the protein in intracellular bacteria, and that elimination of the Rli27-*lmo0514* 5′-UTR interaction interfered with the Lmo0514 protein increase while levels for the long transcript isoform remained unchanged. Our findings thus supported the need for Rli27 binding for efficient Lmo0514 translation. Control qPCR experiments in extracellular bacteria showed similar *lmo0514* transcript levels in this mutant series (Fig. 6E), whereas there were no marked changes in Lmo0514 protein levels (Fig. 6F).

**Figure 7:**
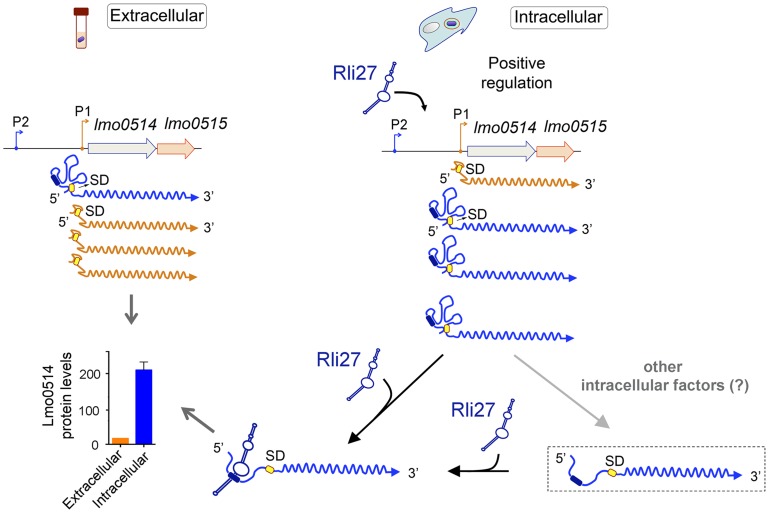
Model of the mechanism by which the *L. monocytogenes* sRNA Rli27 could positively regulate production of the cell wall protein Lmo0514 inside eukaryotic cells. Rli27 production is markedly stimulated in intracellular *L. monocytogenes*. A similar effect is observed for the *lmo0514* long transcript isoform expressed from promoter P2 (blue). Rli27 interaction with the 5′-UTR region located between promoters P1 and P2 could render the Shine-Dalgarno (SD) site (yellow oval) accessible and facilitate Lmo0514 protein translation. The Rli27-binding site is present only in the transcript with the long 5′-UTR (dark blue oval). The model also considers a hypothetical transient state between the occluded and open states of the long 5′-UTR (dashed rectangle). This transient state could be generated by the intervention of other intracellular factors that might help to open or to stabilize the long 5′-UTR for productive ribosome binding and/or Rli27interaction. These putative factors could also promote Lmo0514 translation in the absence of Rli27, as this protein was detected, although at lower levels, in mutant intracellular bacteria that lack this sRNA.

These *in vivo* experiments based on complementation assays with Rli27 variants supported a mechanism that involves Rli27 binding to the 5′-UTR of the long *lmo0514* transcript variant that is upregulated by *L. monocytogenes* inside eukaryotic cells. Such an interaction could promote translation, which would lead to increased Lmo0514 protein levels (Fig. 7).

## Discussion

Given the unique architecture of the cell envelope in Gram-positive bacterial pathogens, cell wall-associated proteins have essential functions in the interplay of these microorganisms with the host [31]. Despite the recognized importance of these proteins in infection, relatively few studies address the spatio-temporal regulation of the production of these proteins following host colonization. Obtaining this information is particularly challenging for Gram-positive pathogens such as *L. monocytogenes* or *Staphylococcus aureus*, which produce a large arsenal of surface proteins with distinct modes of association to the cell wall [31]–[33].

In this study of the Gram-positive bacterium *L. monocytogenes*, we identify sRNA-mediated regulation that acts on a cell wall-associated protein, Lmo0514, during the infection process. During the review process of this work, another report showed regulation of *L. monocytogenes* adhesin LapB by the multicopy sRNA LhrC, although this regulation was not studied in the context of infection [23]. Our data for *lmo0514* also distinguish two transcript isoforms with 5′-UTR of distinct length that are expressed differentially when the pathogen transits between non-host and host environments. These findings are consistent with a regulatory role for the sRNA Rli27, based on its exclusive binding to the *lmo0514* long 5′-UTR variant. This long 5′-UTR is generated from a promoter, here termed P2, which must respond to environmental cues of the eukaryotic intracellular niche. The regulator itself, Rli27, is also upregulated by *L. monocytogenes* following entry into host cells. Transcriptional regulators of *L. monocytogenes* that operate in intracellular bacteria include the alternative sigma factor SigB and the *Listeria*-specific virulence regulator PrfA. Transcriptomic analyses in *sigB* and *prfA* mutants grown in laboratory media did not indicate *lmo0514* as a gene regulated by these factors [34]; our results in intracellular bacteria were also negative (Fig. S5A). A yet undetermined regulator might thus be involved in enhancing transcription from the *lmo0514* P2 promoter. Neither SigB nor PrfA appear to upregulate Rli27 in intracellular bacteria, as determined by real-time qPCR in *sigB* and *prfA* mutants isolated from infected epithelial cells (Fig. S5B).

Comparative transcriptomic studies of *L. monocytogenes* and *L. innocua* show that ∼87% of the genes are transcribed with 5′-UTR shorter than 100 nt, whereas there is a subgroup of approximately 100 genes with long 5′-UTR (>100 nt) [18]; this subgroup includes virulence-related genes and genes with riboswitches [17]. Similar distribution of 5′-UTR length was also described in the related model organism *Bacillus subtilis* [35]. About 80 genes shared by *L. monocytogenes* and *L. innocua* are produced with different-length 5′-UTR [18], which might indicate differences in post-transcriptional regulation of these transcripts. Our data imply a third group of genes based on distinct transcript isoforms that differ in 5′-UTR length. *lmo0514* is a representative example, as it is expressed as two isoforms with 28- and 234-nt 5′-UTR in extra- and intracellular bacteria, respectively. A close parallel is found in a recent work that analyzed sRNA RydC regulation of the *Salmonella enterica cfa* gene, which encodes a cyclopropane fatty acid synthase [36]. RydC selectively stabilizes the longer of two *cfa* transcript isoforms, which is associated to the activity of a distal promoter controlled by σ^A^ and a proximal promoter modulated by σ^B^ [36]. Unlike *lmo0514*, both *cfa* isoforms are expressed by *S. enterica* growing extracellularly in laboratory media. These observations indicate that transcript isoforms with distinct 5′-UTR target platforms for sRNA-mediated post-transcriptional regulation could profoundly influence protein production. It is noteworthy that long 5′-UTRs are frequently associated with genes involved in pathogenesis [18], [37].

An interesting feature predicted by the Mfold program is that Rli27 binding to the *lmo0514* long 5′-UTR could expose the Shine-Dalgarno site, in contrast to the occluded configuration predicted when this 5′-UTR folds as single molecule (Fig. S6, S7). This led us to propose that Rli27 positively regulates Lmo0514 protein levels by altering the long 5′-UTR conformation. This mechanism resembles that of the translational regulation of the *rpoS* transcript in *Escherichia coli* [38]. The Shine-Dalgarno site is blocked by a stem-loop in the *rpoS* 5′-UTR, which is released by base pairing of three distinct Hfq-binding sRNA to the same region. Other paradigmatic cases in *L. monocytogenes* include the virulence regulator *prfA, actA*, and the hemolysin (*hly*) genes [25], [39]–[41]. Our hypothesis for *lmo0514* implies that its 234-nt 5′-UTR has considerable secondary structural complexity in the absence of Rli27. This assumption is consistent with the study by Wurtzel et al. [18], in which RNA-seq did not define the *lmo0514* transcriptional start site, although 2018 such sites were mapped in the *L. monocytogenes* genome, which account for 88% of all annotated transcriptional units. Our tentative model (Fig. 7) also considers the *lmo0514* transcript as ‘low-efficiency’ in terms of translation; there are marked differences in Lmo0514 protein levels in bacteria isolated from epithelial cells (>200-fold increase) that are not reflected at the transcript level. The secondary structure prediction for the short (28-nt) 5′-UTR of the extracellular *lmo0514* isoform also suggests probable occlusion of the Shine-Dalgarno site (Fig. S8). Further work is needed to clarify the extent to which such potential structural changes in the 5′-UTR might explain Rli27-mediated regulation.

Our EMSA data infer direct Rli27-5′-UTR-*lmo0514* interaction, which was also relevant *in vivo*, based on data obtained with the Rli27-mut3 variant. This variant did not restore the Lmo0514 protein levels produced by intracellular bacteria (Fig. 6D). We did not obtain perfect complementation with compensatory mutations in the predicted interacting regions, which allows other interpretations. For example, the targetRNA program might have predicted an incorrect pairing site, pairing between the two molecules might require additional factors with a precise stoichiometry, or the *lmo0514* transcript could undergo alternative post-transcriptional regulation; future work will address these possibilities.

We designed *in vivo* experiments to assess the *lmo0514* long 5′-UTR requirement in Lmo0514 protein production in the cell wall of bacteria located inside eukaryotic cells. We tested strains that bear chromosomal mutations in the *lmo0514* long 5′-UTR predicted interaction site or that lack most of the 5′-UTR upstream of the P1 promoter −10 and −35 sites (Fig. S9A, S9B). Lmo0514 protein levels dropped markedly inside the eukaryotic cells for some these mutants, especially in that lacking the *lmo0514* 5′-UTR (Fig. S9C, S9D). Nonetheless, *lmo0514* transcript levels were affected in these mutants in both extra- and intracellular conditions (Fig. S9C, S9D). Due to the clear side effect of the mutations on transcription, these findings remained inconclusive.

In summary, our results demonstrate that Rli27 is a regulatory sRNA in *L. monocytogenes*, with an essential role as a positive regulator of the Lmo0514 surface protein during the intracellular infection cycle. We also provide evidence that the Rli27 regulatory role is directed to a transcript isoform that bears the binding site for this sRNA; in addition, we show that this isoform is specifically upregulated by intracellular bacteria. Further research will be necessary to determine how Rli27 might modify the secondary structure of the 5′-UTR after binding, and whether such a role requires additional factors also probably upregulated in intracellular bacteria. Another challenge will be to identify the host-derived signal that triggers transcription from the P2 promoter in intracellular *L. monocytogenes* and the bacterial transcriptional factor responsible.

## Materials and Methods

### Comparative genomics

To compare the genome region bearing *rli27* in *L. monocytogenes* EGD-e, *L. innocua* Clip11262 and *L. welshimeri* serovar 6b str. SLCC5334, we used the WEBACT program (http://www.webact.org/WebACT/home). Genome sequences were obtained from the Genbank repository (http://www.ncbi.nlm.nih.gov/genbank/) with entry numbers NC_003210.1, NC_003212.1 and NC_008555.1 for *L. monocytogenes* EGD-e, *L. innocua* Clip11262 and *L. welshimeri* serovar 6b str. SLCC5334, respectively.

### Bacterial strains and growth conditions

The *L. monocytogenes* strains of serotype 1/2a used here are isogenic to wild-type strain EGD-e [27] (listed in Table S1). For sRNA overexpression analyses, the *rli27* wild-type allele was cloned in the pP1 plasmid [42] using Lmorli27-pP1-F and Lmorli27-pP1-R primers (Table S2). Relative expression of cloned sRNA was monitored by semi-quantitative RT-PCR using Lmorli27-F and Lmorli27-R primers (Table S2). *L. monocytogenes* strains were grown at 37°C in brain heart infusion (BHI) broth. For cloning, *E. coli* strains were grown in Luria Bertani (LB) broth at 37°C. When appropriate, media were supplemented with erythromycin (1.5 µg/ml) or ampicillin (100 µg/ml).

### Generation of Rli27 variants for overexpression in *in vivo* assays

Two Rli27 variants, Rli27-mut1 and Rli27-mut3, were constructed by amplification of the *rli27* gene with degenerate primers Lmorli27-pP1-F-mut1 and Lmorli27-pP1-F-mut3 (Table S2) and subsequent cloning in pP1 plasmid [42]. The mut1 mutation introduces 3 nt changes and mut3, 14 nt changes in the major predicted interaction site (see Fig. 5B).

### Construction of Rli27-defective *L. monocytogenes* mutants

To generate the Δ*rli27* mutant strain, fragments of ∼500-bp DNA flanking *rli27* were amplified by PCR using chromosomal DNA of *L. monocytogenes* strain EGD-e and cloned into the thermo-sensitive suicide integrative vector pMAD [43] with primers Lmorli27-A, Lmorli27B, Lmorli27-C and Lmorli27-D (Table S2). Genes were deleted by double recombination as described [43], and deletion was verified by PCR. To generate the Δ*rli27* mutant, we left 9 nt in the 5′ end and 50 nt in the 3′ end of the *rli27* gene, to avoid interference with the *lmo0412* terminator (shared with *rli27*) and the *lmo0411* predicted promoter sequence (Fig. S4). This Δ*rli27* mutation affected *lmo0411* transcript levels slightly. A new deletion mutant was generated (Δ*rli27C2T*), which retains a 5′ extended region of the predicted *lmo0411* promoter, thus maintaining 21 nt in the 5′ end and 50 nt in the 3′ end of *rli27* (Fig. S4). In addition, a strong artificial terminator sequence between the remaining *rli27* sequences was introduced in the Δ*rli27C2T* mutant (Fig. S4). All deletions were confirmed by PCR and sequencing, using primers listed in Table S2.

### Construction of *L. monocytogenes* mutants with chromosomal mutations in the *lmo0514* 5′-UTR

Three types of mutants were constructed with the following chromosomal mutations: i) changes in 3 nt of the long 5′-UTR-*lmo0514* to compensate the mutation in Rli27-mut1 (see Fig. S3, S9), ii) changes in 14 nt of the long 5′-UTR-*lmo0514* to compensate the mutation in Rli27-mut3 (see Fig. S3, S9), and iii) a 174-nt deletion upstream of the −10 and −35 sites of the P1 *lmo0514* promoter (Fig. S9). These changes were generated by double recombination as described [43] and when required, using overlapping SOEing PCR. The oligonucleotide primers for these procedures included Δ0514_P2_A, Δ0514_P2_B, Δ0514_P2_C, Δ0514_P2_D, Mut0514pXG_1-overlap, Mut0514pXG_2-overlap, Mut0514pXG_5-overlap and Mut0514pXG_6-overlap (Table S2).

### Isolation of intracellular bacteria for RNA expression and proteomic analyses

Intracellular bacteria were collected from the human epithelial cell line JEG-3 at 6 h post-infection, as described [12]. For total RNA isolation, epithelial cells cultured in BioDish-XL plates (351040, BD Biosciences) at ∼80% confluence (∼5.6×10^7^ cells) were infected (30 min) with *L. monocytogenes* grown in BHI medium (37°C, overnight) in static non-shaking conditions. RNA was purified using the TRIzol reagent method [17]. For cell wall protein analysis, intracellular bacteria were obtained from JEG-3 cells cultured on four BioDish-XL plates and infected for 6 h [12]. Subcellular fractions containing protoplasts and peptidoglycan-associated proteins were obtained by mutanolysin treatment of intact bacteria as described [10], [12], except that bacterial pellets were incubated for 5 h in lysis buffer (10 mM Tris HCl pH 6.9, 10 mM MgCl_2_, 0.5 M sucrose, 60 µg/ml mutanolysin, 250 µg/ml RNAse-A, 1× protease inhibitor).

### Bacterial fractionation and western blot analysis

Subcellular fractions containing protoplasts and cell wall-associated proteins of *L. monocytogenes* grown at 37°C in BHI media were obtained as described [10]. A volume of protoplasts and the cell wall fraction was analyzed by SDS-PAGE followed by Western blot using *B. subtilis* RecA-specific rabbit polyclonal antibody (a gift of Dr. JC Alonso, Centro Nacional de Biotecnología-CSIC) and rabbit poyclonal sera to the *L. monocytogenes* LPXTG surface proteins Lmo0263 (InlH), Lmo0433 (InlA) and Lmo0514 [12]. RecA (for the protoplast fraction) and LPXTG proteins InlA and InlH (for the cell wall fraction) were used as loading controls. Goat anti-rabbit antibodies conjugated to horseradish peroxidase (Bio-Rad) were used as secondary antibodies. Proteins were visualized by chemoluminescence using luciferin-luminol reagents.

### RNA preparation and reverse transcriptase PCR assays

Total RNA from extracellular bacteria grown to exponential (OD_600_ ∼0.2) and non-shaking stationary phase (OD_600_ ∼1.0) was prepared as described [11]. Oligonucleotides for RT-PCR assays were designed using Primer Express v3.0 (Applied Biosystems)(listed in Table S2). RNA was treated with DNase I (Turbo DNA-free kit, Ambion/Applied Biosystems) at 37°C for 30 min. RNA integrity was assessed by agarose-TAE electrophoresis. RT-PCR was performed using the one-step RT-PCR kit (Qiagen). Briefly, RT-PCR were carried out with 10 to 70 ng RNA (depending on the gene analyzed) in the following conditions: 50°C for 35 min, 95°C for 15 min, followed by 30 cycles (16 cycles for the 16S rRNA gene) of 94°C for 30 s, 55°C for 30 s, and 72°C for 1 min, and then an additional elongation step at 72°C for 10 min. The gene that encodes 16S rRNA was used as a housekeeping gene for all strains in all experimental conditions [44].

### cDNA libraries and real-time quantitative PCR (qPCR)

For cDNA library construction, we used 1 µg of total DNA-free RNA and the High-Capacity cDNA Archive kit (Applied Biosystems) including a random hexamer mix. Reverse transcription was performed at a one-step run of 25°C for 10 min, 37°C for 2 h and 85°C for 5 min. Primers for qPCR were designed using Primer3 [45](listed in Table S2). qPCR was performed in a 10 µl final volume with 1 ng of the cDNA library as template, 500 nM of gene-specific primers and the Power SYBR Green PCR Master Mix (Applied Biosystems). Reactions and data analysis were carried out as described [46].

### 5′-rapid amplification of cDNA ends (5′-RACE)

5′-RACE was performed as described [47], with minor modifications. To convert 5′triphosphates to monophosphates, 15 µg DNA-free RNA, isolated from *L. monocytogenes* growing extracellularly at 37°C to stationary phase or from intracellular bacteria collected at 6 h post-infection of epithelial cells, was treated with 25 U tobacco acid pyrophosphatase (TAP) (Epicentre Technologies) at 37°C for 60 min in a total reaction volume of 50 µl containing 50 mM sodium acetate (pH 6.0), 1 mM EDTA, 0.1% β-mercaptoethanol, 0.01% (v/v) Triton X-100 and 80 U RNAsin (Promega). TAP-negative (TAP−) control RNA was processed in the same conditions in the absence of TAP. Following TAP treatment, RNA was phenol/chloroform-extracted and precipitated with sodium acetate and ethanol. Pellets were rinsed with 70% ethanol in DEPC-dH_2_O, then resuspended in 65 µl DEPC-dH_2_O; 29 µl of these TAP+ or TAP-treated RNA were combined with 5.5 µl 10× buffer, 120 U RNasin, 10% (v/v) dimethylsulfoxide, 70 U RNA ligase, 150 µM ATP and 150 ng RNA oligonucleotide adapter, in a total reaction volume of 55 µl. Samples were denatured (95°C, 5 min) and then chilled on ice. RNA adapter ligation was performed (17°C, 12 h). Following ligation, RNA was phenol/chloroform-extracted and converted to cDNA with a *lmo0514*-specific primer (Lmo0514-Pe-3rv) and the Thermoscript RT System (Invitrogen). Reverse transcription was performed in three cycles (55°C, 60°C and 65°C; 20 min each), followed by RNAseH treatment (37°C, 20 min). *lmo0514* cDNA (2 µl) was amplified by PCR with oligonucleotides RaceIN and lmo0514-PE-1rv (30 cycles of 95°C for 15 s, 55°C for 30 s, and 72°C for 1 min), or with oligonucleotides RaceIN and lmo0514-PE-6rv in the same cycling conditions. PCR products were resolved on 2% agarose gels and bands of interest were excised and subcloned into pCR 2.1 TOPO-vector (Invitrogen). Plasmids containing inserts were purified using the QIAprep Spin Miniprep Kit (QIAgen) and sequenced.

### Northern blot assays

To detect the sRNA Rli27 and the 5S rRNA, 15 µg total RNA were electrophoresed in a 6% polyacrylamide 8 M urea gel (1 h, 200 V in 1× TBE). RNA was transferred to a Hybond membrane (Amersham) for 2.5 h at 40 V in 0.5× TBE at 4°C and RNA was UV-crosslinked to the membrane. Membranes were pre-hybridized with UltraHyb buffer (Ambion; 65°C, 2 h) and hybridized with 10^6^ cpm ^32^P-labeled specific riboprobes (65°C, overnight). Membranes were washed with 2× SSC, 0.5% SDS and 1× SSC, 0.1% SDS and exposed to X-ray film. 5S rRNA was used as control [22]. A nonradioactive digoxigenin (DIG)-based RNA detection protocol was used for Northern blot analysis of *lmo0514* and the 16S rRNA. Total RNA (1 µg for *lmo0514* or 200 ng for 16S rRNA) was separated on a 1.5% agarose denaturing gel (2% formaldehyde, 1× MOPS), overnight capillary transferred to a Hybond membrane in 20× SSC, and UV-crosslinked. The membrane was prehybridized (68°C, 1 h) and then hybridized with DIG-labeled *lmo0514* and 16S rRNA probes (68°C, overnight). Immunological detection of RNA was performed (DIG Northern starter kit; Roche) and exposed to X-ray film.

### Electrophoretic mobility shift assays (EMSA)

Gel mobility shift assays were performed with 1.48 pmol *in vitro*-transcribed RNA corresponding to the *lmo0514* 5′-UTR (nucleotides −234 to −14 from the *lmo0514* ATG codon) and increasing concentrations of *in vitro*-transcribed RNAs for Rli27 wild-type, Rli27-mut1 and Rli27-mut3. These *in vitro*-transcribed molecules included Rli27 nucleotides 1 to 131 plus an additional 60 nt at the 3′ end, as designed for optimal amplification. We produced *lmo0514* 5′-UTR variants with compensatory mutations in 3 nt (mut-1) or 14 nt (mut-3) for those generated in Rli27. Oligonucleotide primers used are listed in Table S2. We also generated an amplified molecule corresponding to RNA SbrA (Rli11) encompassed nucleotides 1 to 69 of the total of 71 nucleotides. The reaction was carried out in 10 µl of 1× binding buffer (20 mM Tris-acetate pH 7.6, 100 mM sodium acetate, 5 mM magnesium acetate, 20 mM EDTA) (37°C, 1 h). The binding reactions were mixed with 2 µl loading dye (48% glycerol, 0.01% orange G) and loaded on native 4% polyacrylamide gels, followed by electrophoresis in 0.5× TBE buffer (200 V, 4°C). Gels were stained with Gel Red nucleic acid stain (Biotium) and photographed under UV transillumination with the GelDoc 2000 system (Bio-Rad).

### Computational prediction of potential interactors with the *lmo0514* long 5′-UTR region

The bioinformatic program TargetRNA (http://cs.wellesley.edu/~btjaden/TargetRNA2/) [28] was used to predict non-coding RNAs that could bind to the *lmo0514* long 5′-UTR (234 nt from the ATG codon). Predictive folding of the *lmo0514* long 5′-UTR alone or with sRNA Rli27 was done using Mfold (http://mfold.rna.albany.edu/?q=mfold).

### Statistical and densitometry analyses

Statistical significance was analyzed with GraphPad Prism v5.0b software (GraphPad Inc.) using Student's *t*-test. A *P* value≤0.05 was considered significant. For densitometry of bands obtained in western blots, we used ImageJ software (National Institutes of Health of USA [http://imagej.nih.gov/ij/]).

## Supporting Information

Figure S1 *lmo0514* is cotranscribed with the downstream gene *lmo0515*, which codes for a universal stress protein in *L. monocytogenes* [26]. PCR assays performed on reverse-transcribed RNA (cDNA) and genomic DNA (gDNA). C(-) refers to a control sample that lacks a template. Colors indicate relative position of the Lmo0514-F and 0515-R primers (Table S2). RNA and DNA were isolated from *L. monocytogenes* wild-type strain EGD-e (WT) grown to stationary phase (OD_600_ ∼1.0) at 37°C in BHI medium in non-shaking conditions.
(TIF)

Figure S2 Comparison of the *rli27* region of *L. monocytogenes* EGD-e strain (Lmo) with the respective regions of non-pathogenic species *L. innocua* (Lin) and *L. welshimeri* (Lwe). Genomes were compared using the WebACT tool (http://www.webact.org/WebACT/home). Red indicates similar genomic organization; blue indicates inversions. Orthologous genes are shown in same color. The *rli27* gene of *L. monocytogenes* has no ortholog in *L. welshimeri* and is flanked by *lmo0411* and *lmo0412*, two genes in the opposite DNA strand predicted to encode a protein similar to phosphoenolpyruvate synthase and a protein of unknown function, respectively.
(TIF)

Figure S3 Scheme of all predicted interaction sites between Rli27 and the *lmo0514* 5′-UTR. Interactions were predicted using the targetRNA program (http://cs.wellesley.edu/~btjaden/TargetRNA2/). Scheme shows the exact positions of the predicted interaction regions between the *lmo0514* 5′-UTR and Rli27 as well as the mutant variants Rli27-mut1 and Rli27-mut3, with changes highlighted in yellow. Note that the hybridization energy is lower in the case of the Rli27 variants. Compensatory mutant variants of the 5′-UTR-*lmo0514* molecule (*lmo0514-mut1*, *lmo0514*-mut3) are also shown.
(TIF)

Figure S4 Genome region of the *L. monocytogenes* EGD-e strain bearing *rli27* and its flanking genes, and the exact location of the deletions generated in the Δ*rli27* and Δ*rli27C2T* mutants. Blue boxes represent the predicted −10 and −35 sites of the *lmo0411* promoter. Note that a strong artificial terminator was introduced in Δ*rli27C2T* to avoid expression of the remaining Rli27-specific sequences. Both *rli27* and *lmo0412* genes share a Rho-independent terminator.
(TIF)

Figure S5 *lmo0514* and *rli27* expression are not regulated by SigB or PrfA in extra- or intracellular bacteria. (A) qPCR data relative to *lmo0514* obtained from total RNA isolated from extracellular bacteria grown in BHI medium to stationary phase (extracellular) or collected from epithelial cells (intracellular). Primers Utr0514_qPCR_F and Utr0514_qPCR_R were used. (B) Data relative to Rli27 expression. No significant differences were found for any of the samples. Data are derived from a minimum of three independent experiments.
(TIF)

Figure S6 Energetically favorable conformation of the *lmo0514* long 5′-UTR (234 nt) as single molecule, as predicted by the M-fold program (http://mfold.rna.albany.edu/?q=mfold). Note that the Shine-Dalgarno site appears to be occluded.
(TIF)

Figure S7 Energetically favorable conformation of the *lmo0514* long 5′-UTR (234 nt) combined with Rli27, as predicted by M-fold (http://mfold.rna.albany.edu/?q=mfold). Note the opening of the Shine-Dalgarno (SD) site.
(TIF)

Figure S8 Energetically favorable conformation of the *lmo0514* short 5′-UTR (28 nt) as a single molecule, predicted by M-fold (http://mfold.rna.albany.edu/?q=mfold). Note the occlusion of the Shine-Dalgarno (SD) site.
(TIF)

Figure S9 *In vivo* experiments using *L. monocytogenes* mutants with chromosomal mutations in the *lmo0514* 5′-UTR. (A) Detail of the mut1 and mut3 chromosomal mutations introduced in the *lmo0514* 5′-UTR. These mutations were designed to compensate the mut1 and mut3 mutations generated in the Rli27 variants. (B) Scheme of the mutations introduced in the chromosome: Δ5′-UTR-*lmo0514* (174-nt deletion), 5′-UTR-mut1 (3-nt change) and 5′-UTR-mut3 (14-nt change). (C) Effect of these chromosomal mutations on Lmo0514 protein and *lmo0514* ORF levels in extracellular bacteria grown in BHI medium to stationary phase. (D) Effect of these chromosomal mutations on Lmo0514 protein and *lmo0514* transcript levels (differentiating production of the long 5′-UTR isoform) in intracellular bacteria collected from epithelial cells. Note the marked decrease in Lmo0514 protein by the Δ5′-UTR-*lmo0514* and the 5′-UTR-mut3 mutants in the intracellular niche of the eukaryotic cell. These mutants nonetheless have low *lmo0514* transcript expression, probably due to side effects linked to loss of the 5′-UTR region between the P2 and P1 promoters.
(TIF)

Table S1 *Listeria monocytogenes* strains used in this study.
(PDF)

Table S2 Oligonucleotide primers used in this study.
(PDF)
