# Supplementary figures and images for: The Listeria Small RNA Rli27 Regulates a Cell Wall Protein inside Eukaryotic Cells by Targeting a Long 5′-UTR Variant
Source: PLoS Genet. 2014 Oct 30;10(10):e1004765. doi: 10.1371/journal.pgen.1004765 (PMC4214639; doi:10.1371/journal.pgen.1004765)

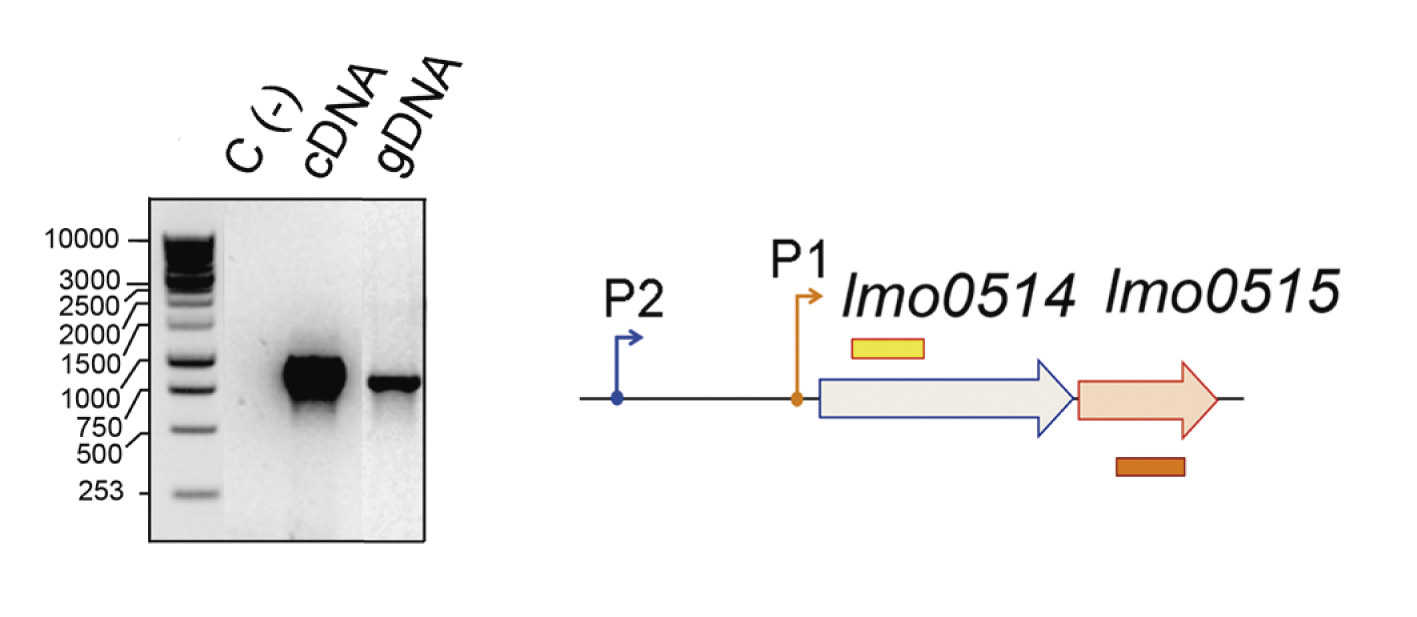

Supplement: Figure S1 — lmo0514 is cotranscribed with the downstream gene lmo0515, which codes for a universal stress protein in L. monocytogenes [26]. PCR assays performed on reverse-transcribed RNA (cDNA) and genomic DNA (gDNA). C(-) refers to a control sample that lacks a template. Colors indicate relative position of the Lmo0514-F and 0515-R primers (Table S2). RNA and DNA were isolated from L. monocytogenes wild-type strain EGD-e (WT) grown to stationary phase (OD600 ∼1.0) at 37°C in BHI medium in non-shaking conditions. (TIF) [file pgen.1004765.s001.tif]

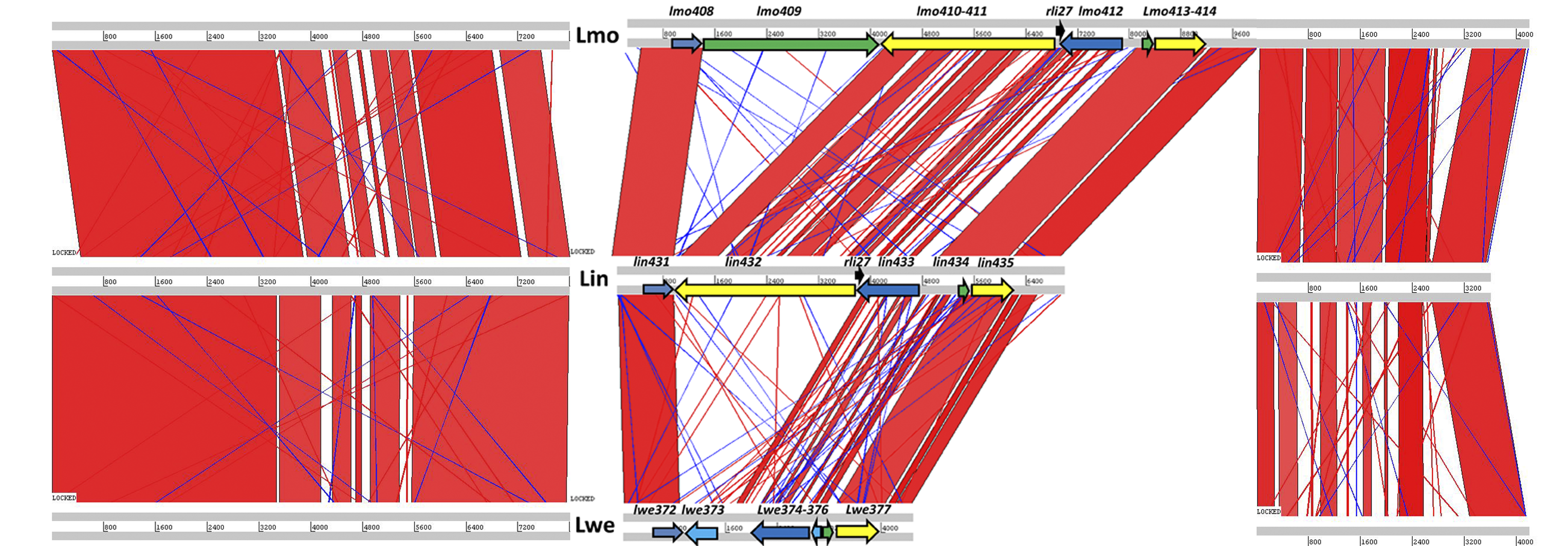

Supplement: Figure S2 — Comparison of the rli27 region of L. monocytogenes EGD-e strain (Lmo) with the respective regions of non-pathogenic species L. innocua (Lin) and L. welshimeri (Lwe). Genomes were compared using the WebACT tool (http://www.webact.org/WebACT/home). Red indicates similar genomic organization; blue indicates inversions. Orthologous genes are shown in same color. The rli27 gene of L. monocytogenes has no ortholog in L. welshimeri and is flanked by lmo0411 and lmo0412, two genes in the opposite DNA strand predicted to encode a protein similar to phosphoenolpyruvate synthase and a protein of unknown function, respectively. (TIF) [file pgen.1004765.s002.tif]

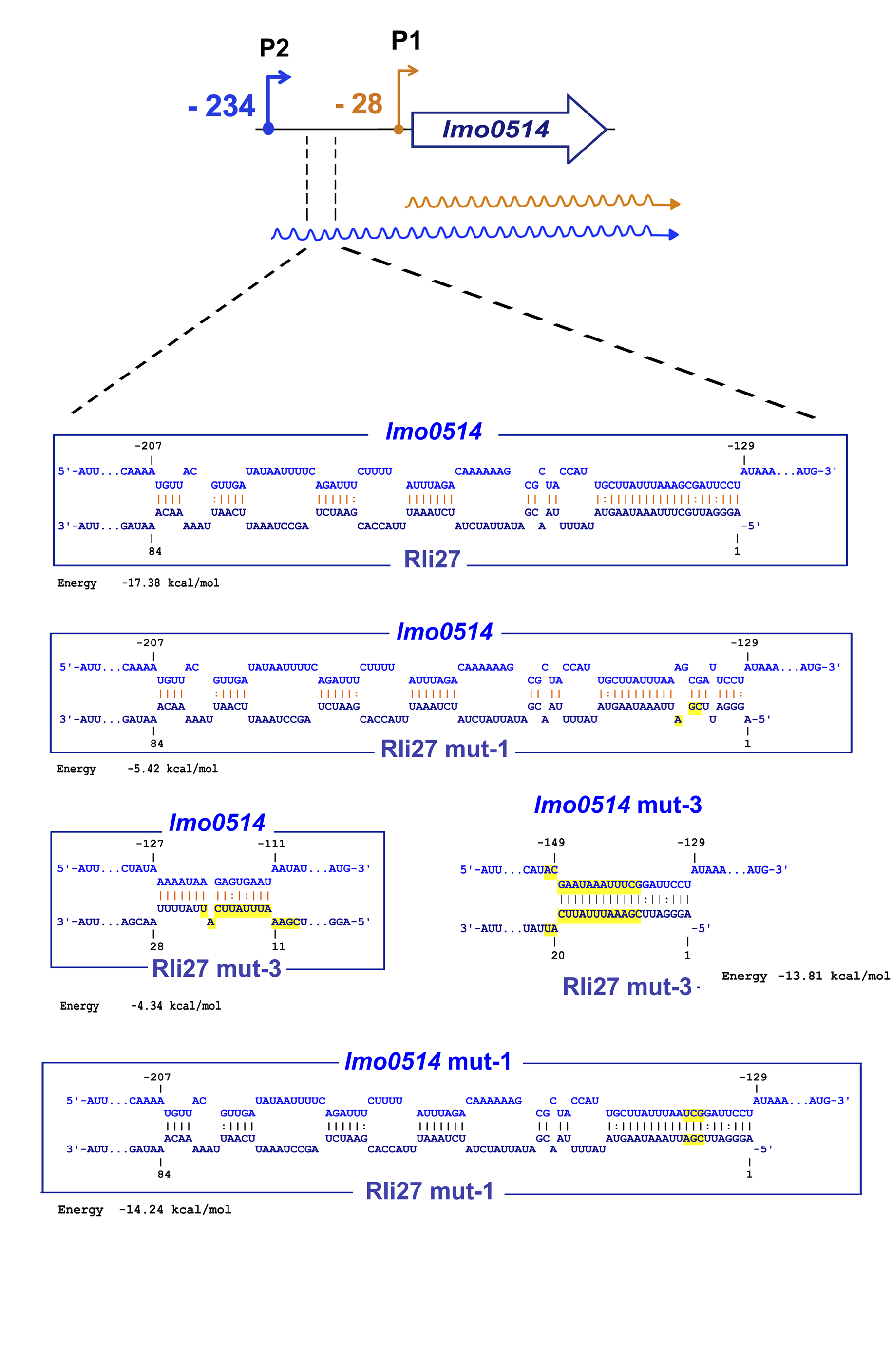

Supplement: Figure S3 — Scheme of all predicted interaction sites between Rli27 and the lmo0514 5′-UTR. Interactions were predicted using the targetRNA program (http://cs.wellesley.edu/~btjaden/TargetRNA2/). Scheme shows the exact positions of the predicted interaction regions between the lmo0514 5′-UTR and Rli27 as well as the mutant variants Rli27-mut1 and Rli27-mut3, with changes highlighted in yellow. Note that the hybridization energy is lower in the case of the Rli27 variants. Compensatory mutant variants of the 5′-UTR-lmo0514 molecule (lmo0514-mut1, lmo0514-mut3) are also shown. (TIF) [file pgen.1004765.s003.tif]

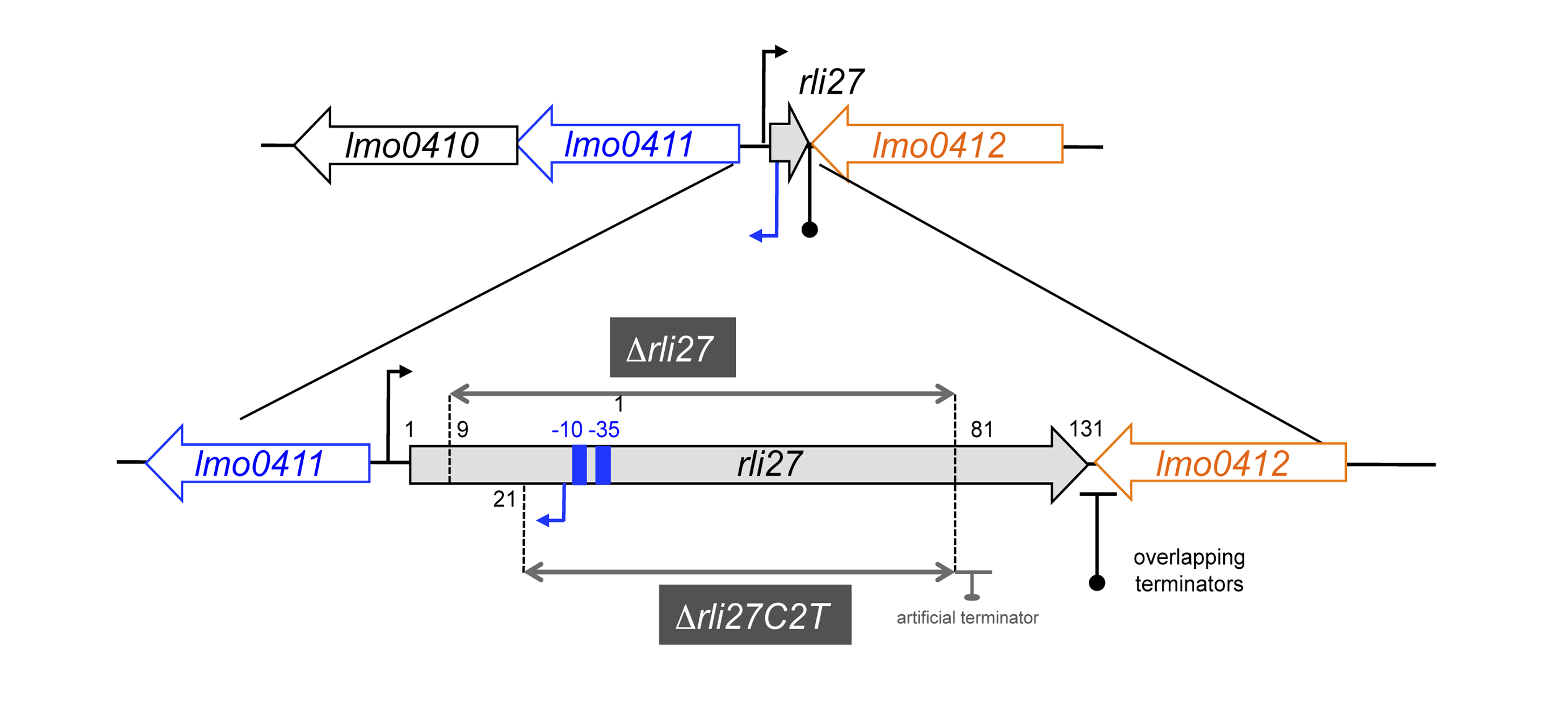

Supplement: Figure S4 — Genome region of the L. monocytogenes EGD-e strain bearing rli27 and its flanking genes, and the exact location of the deletions generated in the Δrli27 and Δrli27C2T mutants. Blue boxes represent the predicted −10 and −35 sites of the lmo0411 promoter. Note that a strong artificial terminator was introduced in Δrli27C2T to avoid expression of the remaining Rli27-specific sequences. Both rli27 and lmo0412 genes share a Rho-independent terminator. (TIF) [file pgen.1004765.s004.tif]

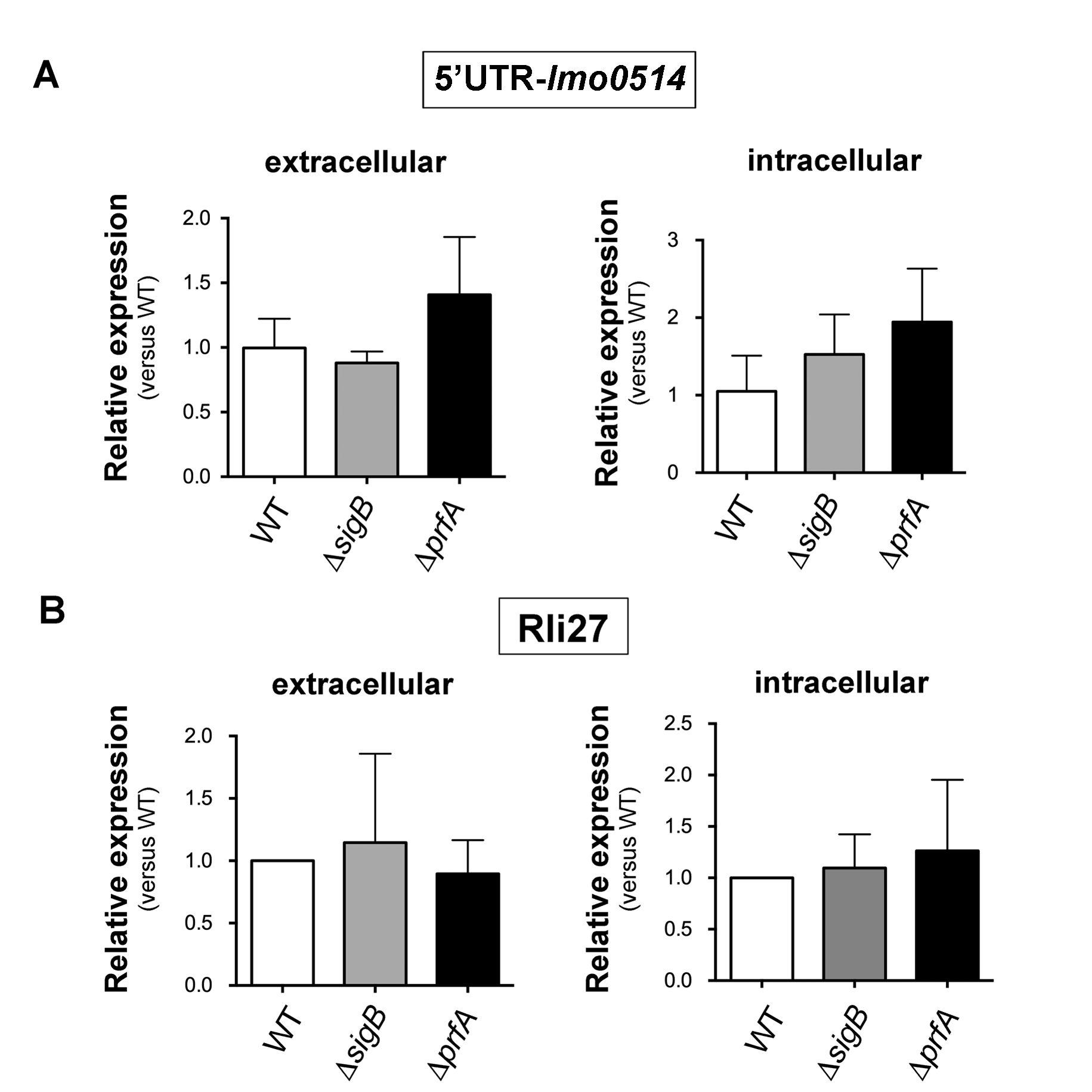

Supplement: Figure S5 — lmo0514 and rli27 expression are not regulated by SigB or PrfA in extra- or intracellular bacteria. (A) qPCR data relative to lmo0514 obtained from total RNA isolated from extracellular bacteria grown in BHI medium to stationary phase (extracellular) or collected from epithelial cells (intracellular). Primers Utr0514_qPCR_F and Utr0514_qPCR_R were used. (B) Data relative to Rli27 expression. No significant differences were found for any of the samples. Data are derived from a minimum of three independent experiments. (TIF) [file pgen.1004765.s005.tif]

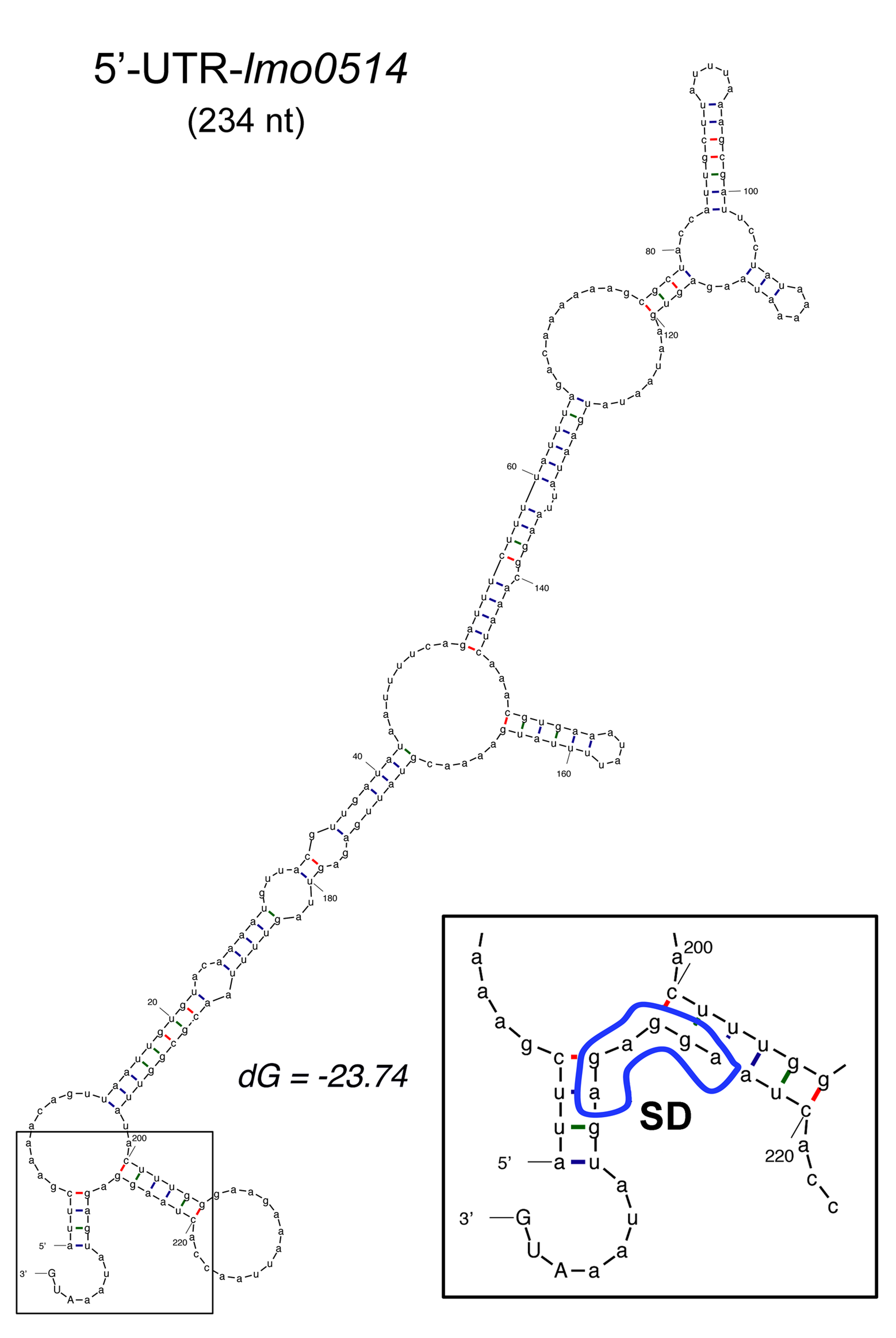

Supplement: Figure S6 — Energetically favorable conformation of the lmo0514 long 5′-UTR (234 nt) as single molecule, as predicted by the M-fold program (http://mfold.rna.albany.edu/?q=mfold). Note that the Shine-Dalgarno site appears to be occluded. (TIF) [file pgen.1004765.s006.tif]

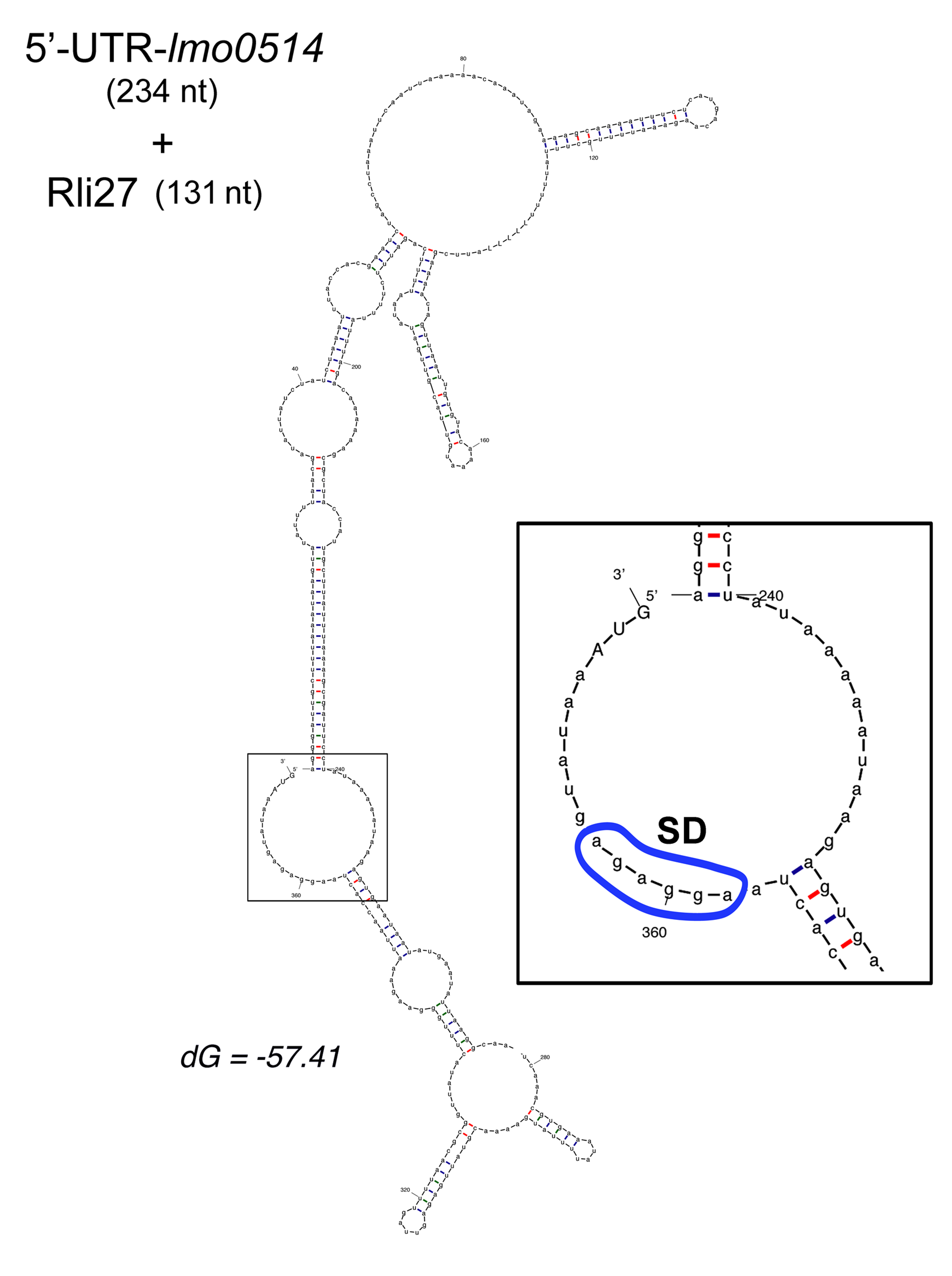

Supplement: Figure S7 — Energetically favorable conformation of the lmo0514 long 5′-UTR (234 nt) combined with Rli27, as predicted by M-fold (http://mfold.rna.albany.edu/?q=mfold). Note the opening of the Shine-Dalgarno (SD) site. (TIF) [file pgen.1004765.s007.tif]

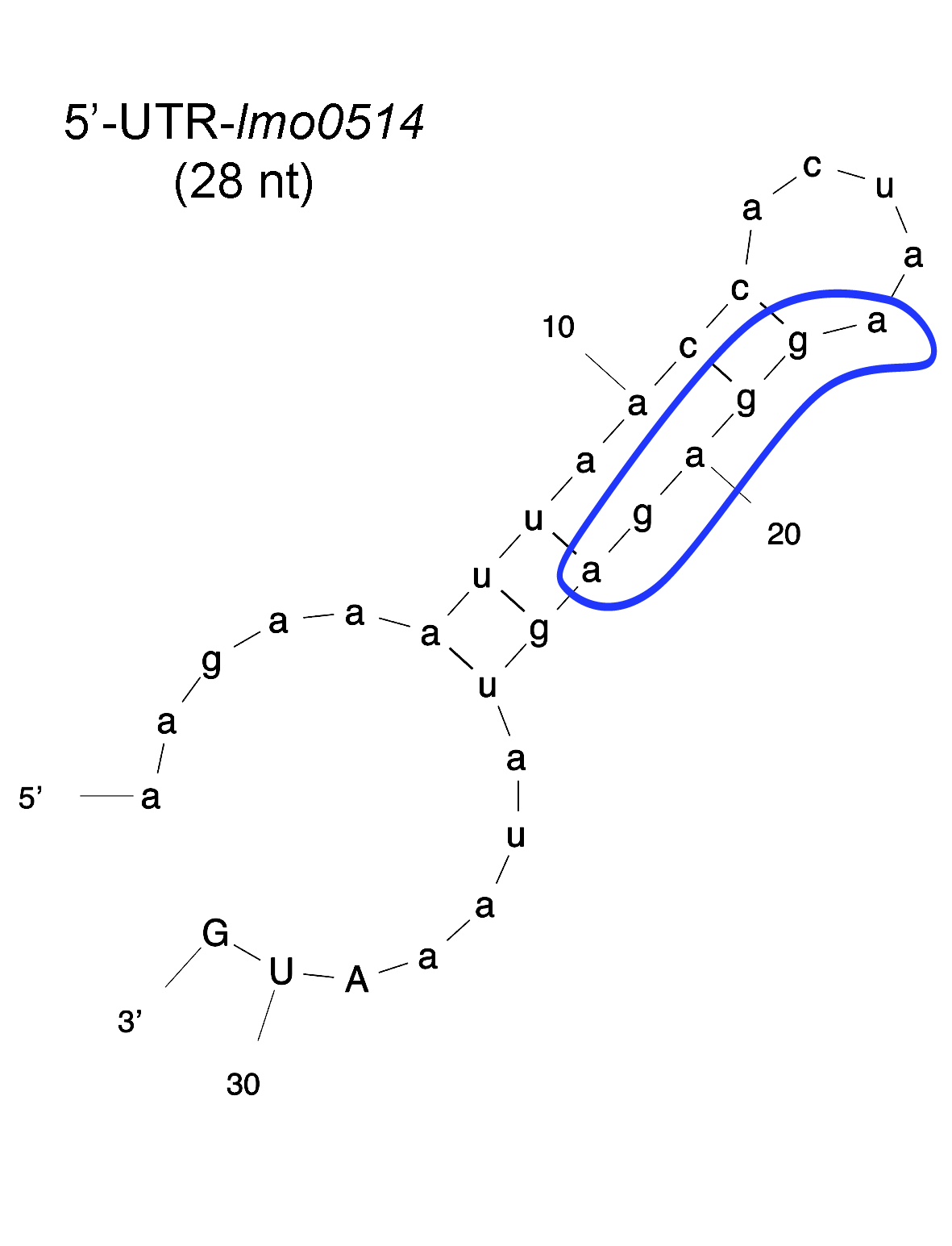

Supplement: Figure S8 — Energetically favorable conformation of the lmo0514 short 5′-UTR (28 nt) as a single molecule, predicted by M-fold (http://mfold.rna.albany.edu/?q=mfold). Note the occlusion of the Shine-Dalgarno (SD) site. (TIF) [file pgen.1004765.s008.tif]

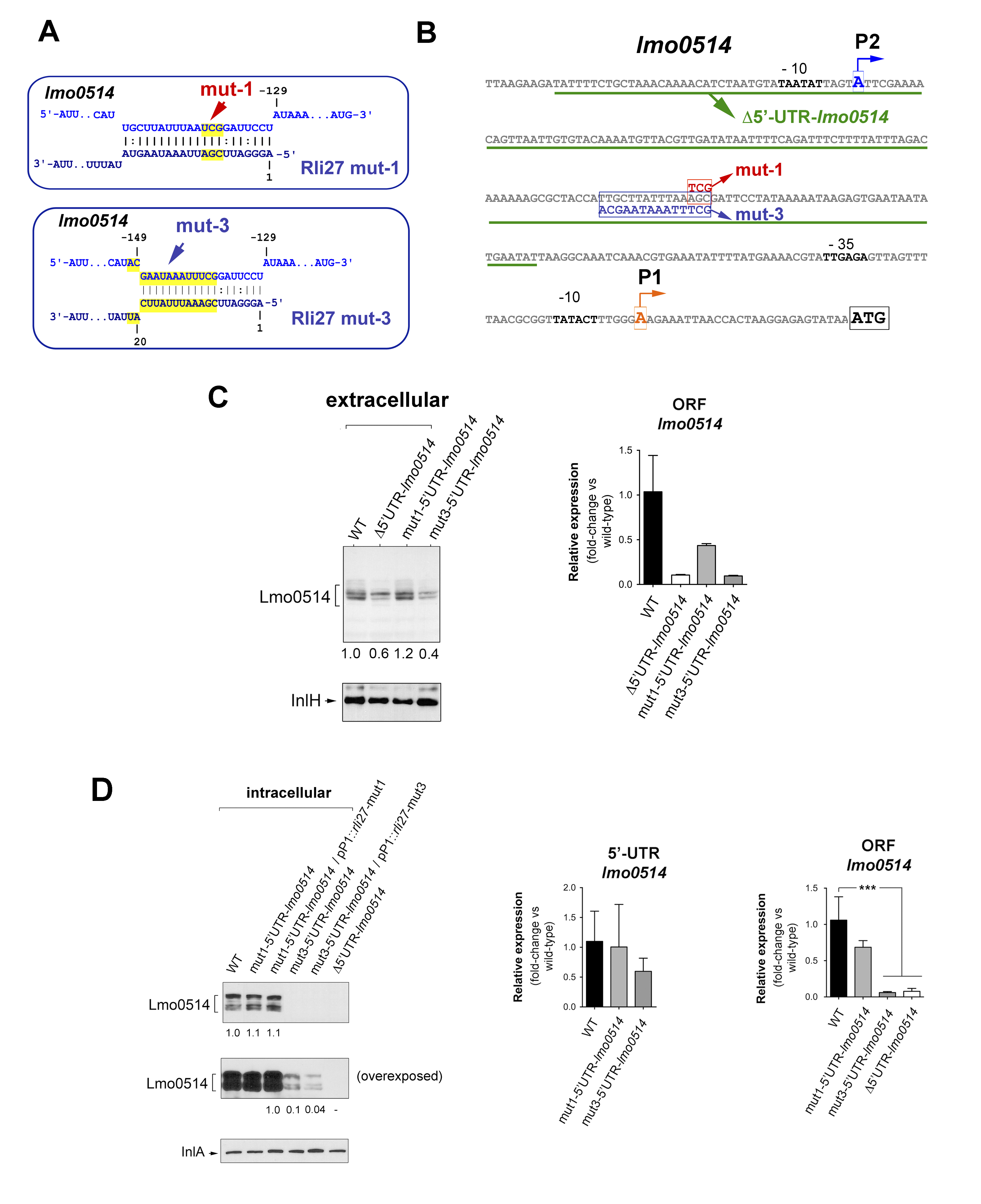

Supplement: Figure S9 — In vivo experiments using L. monocytogenes mutants with chromosomal mutations in the lmo0514 5′-UTR. (A) Detail of the mut1 and mut3 chromosomal mutations introduced in the lmo0514 5′-UTR. These mutations were designed to compensate the mut1 and mut3 mutations generated in the Rli27 variants. (B) Scheme of the mutations introduced in the chromosome: Δ5′-UTR-lmo0514 (174-nt deletion), 5′-UTR-mut1 (3-nt change) and 5′-UTR-mut3 (14-nt change). (C) Effect of these chromosomal mutations on Lmo0514 protein and lmo0514 ORF levels in extracellular bacteria grown in BHI medium to stationary phase. (D) Effect of these chromosomal mutations on Lmo0514 protein and lmo0514 transcript levels (differentiating production of the long 5′-UTR isoform) in intracellular bacteria collected from epithelial cells. Note the marked decrease in Lmo0514 protein by the Δ5′-UTR-lmo0514 and the 5′-UTR-mut3 mutants in the intracellular niche of the eukaryotic cell. These mutants nonetheless have low lmo0514 transcript expression, probably due to side effects linked to loss of the 5′-UTR region between the P2 and P1 promoters. (TIF) [file pgen.1004765.s009.tif]
