# Supplementary material for: The Listeria Small RNA Rli27 Regulates a Cell Wall Protein inside Eukaryotic Cells by Targeting a Long 5′-UTR Variant
Source: PLoS Genet. 2014 Oct 30;10(10):e1004765. doi: 10.1371/journal.pgen.1004765 (PMC4214639; doi:10.1371/journal.pgen.1004765)
Supplement: Table S1 — Listeria monocytogenes strains used in this study. (PDF) [file pgen.1004765.s010.pdf]

Table S1. *Listeria monocytogenes* strains used in this study

Species / strain	Relevant genotype	Source or reference
EGD-e	wild-type	[1]
MD3409	EGD-e / pP1	This work
MD3441	EGD-e / pP1::*rli27*	This work
MD3443	Δ*rli27*	This work
MD3444	Δ*rli27C2T*	This work
MD3460	Δ*rli27* / pP1	This work
MD3461	Δ*rli27C2T* / pP1	This work
MD3457	Δ*rli27* / pP1::*rli27*	This work
MD2356	Δ*rli27C2T* / pP1::*rli27-mut1*	This work
MD2361	Δ*rli27C2T* / pP1::*rli27-mut3*	This work
MD2366	Δ5′-UTR*lmo0514*	This work
MD2358	5′-UTR-*lmo0514-mut1*	This work
MD2359	5′-UTR-*lmo0514-mut3*	This work
MD2360	5′-UTR*lmo0514-mut1* / pP1-*rli27-mut1*	This work
MD2365	5′-UTR*lmo0514-mut3* / pP1-*rli27-mut3*	This work
MD3449	Δ*lmo0411*	This work
MD3402	Δ*lmo0412*	[2]
MD2302	Δ*lmo0514*	Trinad Chakraborty

1. Glaser P, Frangeul L, Buchrieser C, Rusniok C, Amend A, et al. (2001) Comparative genomics of *Listeria* species. Science 294: 849-852.
2. Quereda JJ, Pucciarelli MG (2014) Deletion of the membrane protein Lmo0412 increases the virulence of *Listeria monocytogenes*. Microbes Infect. doi 10.1016/ j.micinf.2014.07.002
