# Supplementary material for: The Listeria Small RNA Rli27 Regulates a Cell Wall Protein inside Eukaryotic Cells by Targeting a Long 5′-UTR Variant
Source: PLoS Genet. 2014 Oct 30;10(10):e1004765. doi: 10.1371/journal.pgen.1004765 (PMC4214639; doi:10.1371/journal.pgen.1004765)
Supplement: Table S2 — Oligonucleotide primers used in this study. (PDF) [file pgen.1004765.s011.pdf]

Table S2. Oligonucleotide primers used in this study

Name/number	Sequence (5′-3′) *	Use
Lmorli27-A	CGCGGATCCCGGAGAAAGTTGATAATACACCTG	Amplification of ~500 bp fragment in 3′ flanking region of *rli27*
Lmorli27-B	AAAAGGCCTCAAATAGAAAAGCAAAATTTCTCA	Amplification of ~500 bp fragment in 3′ flanking region of *rli27*
Lmorli27-C	AAAAGGCCTGCAATCCCTATTTTAATTATACAATAAGTAG	Amplification of ~500 bp fragment in 5′ flanking region of *rli27*
Lmorli27-D	CCGGAATTCCGAAGCCCAACACATACTGA	Amplification of ~500 bp fragment in 5′ flanking region of *rli27*
Lmorli27-C2-term	AAAAGGCCT*AAAAGGCCATCCGTCAGGATGGCCTTCT*TACTTATTTAAAGCAATCCCTATTTT	Amplification of ~500 bp fragment in 5′ flanking region of *rli27* to introduce an artificial terminator
Lmorli27 test-F2	GGTTCGCTTGTAAGCCTCTG	Verification of the Δ*rli27* deletion (mapping in flanking region)
Lmorli27 test-R2	CACCGCTACTGGCAAAAGAT	Verification of the Δ*rli27* deletion (mapping in flanking region)
Lmorli27-pP1-F	CGAGCTCAGGGATTGCTTTAAATAAGTATATTTT	Cloning of *rli27* for overexpression and complementation assays
Lmorli27-pP1-R	ACATGCATGCAAAAATAAAGCAAAATTTCTTGTCATGAG	Cloning of *rli27* for overexpression and complementation assays
Lmorli27-pP1-F-mut1	CGAGCTCAGGGATTCGATTAAATAAGTATATTTTAACGATATTATC	Cloning of a mutated version of *rli27* (mut-1*)* for overexpression and complementation assays
Lmorli27-pP1-F-mut3	CGAGCTCAGGGATTCGAAATTTATTCATTATTTTAACGATATTATCTATCTAAATTTA	Cloning of a mutated version of *rli27* (mut-3*)* for overexpression and complementation assays
Lmorli27-F	AAATTTACCACGAATCTAGCC	Verification of Δ*rli27* deletion (mapping in reading frame), qPCR and Northern Blot probe for Rli27
Lmorli27-R	GAAATTTTGCTTTTCTATTTGTT	Verification of Δ*rli27* deletion (mapping in reading frame) and qPCR
Lmo0411-A	CGCGGATCCGAGAAAAAAGCAGCTGAAGAAAA	Amplification of ~500 bp fragment in 5′ flanking region of *lmo0411*
Lmo0411-B	AAAAGGCCTGGCTGTATTTCTTGAAACTTTAG	Amplification of ~500 bp fragment in 5′ flanking region of *lmo0411*
Lmo0411-C	AAAAGGCCTTGTCGAAAATGCAACTACCG	Amplification of ~500 bp fragment in 3′ flanking region of *lmo0411*
Lmo0411-D	CCGGAATTCGAAGTTGGCGGAACAAAATG	Amplification of ~500 bp fragment in 3′ flanking region of *lmo0411*
Lmo0411 test-F	AAGCAAAGGCACAAGGCTTA	Verification of the Δ*lmo0411* deletion (mapping in flanking region)
Lmo0411 test-R	TGGTATGACGCAGAAGTTGG	Verification of the Δ*lmo0411* deletion (mapping in flanking region)
Lmo0411-F	CTTCGCAGGGCAACATGATA	Verification of the Δ*lmo0411* deletion (mapping in reading frame) and qPCR
Lmo0411-R	ACTGGCATCGATAGAGAGGGATT	Verification of the Δ*lmo0411* deletion (mapping in reading frame) and qPCR
Lmo0514-A	AGAGTTCATTAATTGTAAGTCCAGG	Amplification of ~500 bp fragment in 5′ flanking region of *lmo0514*
Lmo0514-B	TTATACTCTCCTTAGTGGTTAATT	Amplification of ~500 bp fragment in 5′ flanking region of *lmo0514*
Lmo0514-C	ATTATTGTGAATTCATTAGCAAAAA	Amplification of ~500 bp fragment in 3′ flanking region of *lmo0514*
Lmo0514-D	CGCTTTTTTTATCTAGCTATTAAAG	Amplification of ~500 bp fragment in 3′ flanking region of *lmo0514*

Δ0514_P2_A	CGCGGATCCGCGTCACTTCACTTCCGTTG	Amplification of ~500 bp fragment (fragment A-B) for generation of Δ5'-UTR *lmo0514* deletion in chromosome
Δ0514_P2_B	AAAAGGCCTAAGGCAAATCAAACGTGAAA	Amplification of ~500 bp fragment (fragment A-B) for generation of Δ5'-UTR *lmo0514* deletion in chromosome
Δ0514_P2_C	AAAAGGCCTTCTTCTTAATAATGCGAAAAAG	Amplification of ~500 bp fragment (fragment C-D) for generation of Δ5'-UTR *lmo0514* deletion in chromosome
Δ0514_P2_D	CCGGAATTCTATAAAAGCGGCACCTGGTC	Amplification of ~500 bp fragment (fragment C-D) for generation of Δ5'-UTR *lmo0514* deletion in chromosome
Lmo0514 test-F	AAAAAGCGCTACCATTGCTT	Verification of the Δ*lmo0514* deletion (mapping in flanking region)
Lmo0514 test-R	CAATTCCAAGCACACCGTCT	Verification of the Δ*lmo0514* deletion (mapping in flanking region)
Lmo0514-F	TGCTGCAGGACTCAAAGCAA	Verification of the Δ*lmo0514* deletion (mapping in reading frame), qPCR and Northern Blot probe for lmo0514
Lmo0514-R	TGTCCACTGTCGCTTGTAGTCA	Verification of the Δ*lmo0514* deletion (mapping in reading frame) and qPCR
Δ0514_comp_A	CGCGGATCCGCATCACTTGCTTGCTTGAG	Generation of mut-1 and mut-3 chromosomal mutations in 5′-UTR of *lmo0514*
Δ0514_comp_D	CCGGAATTCTTCATGTTTTGCTCATTGTTCA	Generation of mut-1 and mut-3 chromosomal mutations in 5′-UTR of *lmo0514*
Mut0514pXG_1-overlap	AGGAATCCGATTAAATAAGCAATGGTAGCGCTTTTTTGTCTAAATAAAA	Generation of mut-1 chromosomal mutation in 5′-UTR of *lmo0514*
Mut0514pXG_2-overlap	CATTGCTTATTTAATCGGATTCCTATAAAAATAAGAGTGAATAATATGAATATTAAG	Generation of mut-1 chromosomal mutation in 5′-UTR of *lmo0514*
Mut0514pXG_5-overlap	AGGAATCCGAAATTTATTCGTATGGTAGCGCTTTTTTGTCTAAATAAAA	Generation of mut-3 chromosomal mutation in 5′-UTR of *lmo0514*
Mut0514pXG_6-overlap	CATACGAATAAATTTCGGATTCCTATAAAAATAAGAGTGAATAATATGAATATTAAG	Generation of mut-3 chromosomal mutation in 5′-UTR of *lmo0514*
UTR-A	GAGAGTTAGTTTTAACGCGGTTATAC	RT-PCR for *lmo0514* untranslated region
UTR-B	TCTTTTATTTAGACAAAAAAGCGC	RT-PCR for *lmo0514* untranslated region
UTR-1R	TGAGCTGCTGTGGAAGGATATA	RT-PCR for *lmo0514* untranslated region
0515-R	CATTTGGAGAAAAGGCACGTA	RT-PCR for *lmo0514-lmo0515 operon*
Lmo16S rRNA-F	CACACTGGGACTGAGACACG	RT-PCR and qPCR
Lmo16S rRNA-R	CCGTCAGACTTTCGTCCATT	RT-PCR and qPCR
Utr0514_qPCR_F	AAAAAGCGCTACCATTGCTT	qPCR for *lmo0514* 5´untranslated region
Utr0514_qPCR_R	TTTCACGTTTGATTTGCCTTA	qPCR for *lmo0514* 5´untranslated region
RNA adapter	GCUGAUGGCGAUGAAUGAACACUGCGUUUGCUGGCUUUGAUGAAA	5´RACE: RNA ligation to RNA oligo
RaceIN	CGCGGATCCGAACACTGCGTTTGCTGGCTTTGATG	5´RACE

lmo0514-PE-1rv	AGAAATAGTTTCAGTGCTTTTTTCAT	5´RACE
lmo0514-PE-3rv	TGAGCTGCTGTGGAAGGATATA	5´RACE
lmo0514-PE-6rv	CACTCTTATTTTTATAGGAATCGCTTT	5´RACE
T7-rv-rli27	TAATACGACTCACTATAGGGGAAATTTTGCTTTTCTATTTGTT	Northern Blot probe for Rli27
5S rRNA-F	TATGGCGAGAAGGTCACACC	Northern Blot probe for 5S rRNA
T7-5S-rRNA-R	TAATACGACTCACTATAGGGAACTACCATTGGCGCAGAGA	Northern Blot probe for 5S rRNA
T7-Lmo0514-R	TAATACGACTCACTATAGGGTGTCCACTGTCGCTTGTAGTCA	Northern Blot probe for *lmo0514*
16S-northern-F1	TTAGCTAGTTGGTAGGGT	Northern Blot probe for 16s
T7-16S-northern-R1	TAATACGACTCACTATAGGGAATCCGGACAACGCTTGC	Northern Blot probe for 16s
EMSAT7-Rli27-WT-F	TAATACGACTCACTATAGGGAGGGATTGCTTTAAATAAGTATATTTTAAC	In vitro transcription of Rli27 wild type for EMSA assay
EMSA-Rli27-WT-R	ACGGCTGTGACAGTTGCTC	In vitro transcription of Rli27 wild type and Rli27^{mut3} for EMSA assay
EMSAT7-Rli27F-Mut1	TAATACGACTCACTATAGGGAGGGATTCGATTAAATAAGTATATTTTAACGATATTATCTATC	
EMSAT7-Rli27F-Mut3	TAATACGACTCACTATAGGGAGGGATTCGAAATTTATTCATTATTTTAACGATATTATCTATCTAAATTTAC	In vitro transcription of Rli27^{mut3} for EMSA assay
EMSAT7-0514-WT-TSS	TAATACGACTCACTATAGGGATTCGAAAACAGTTAATTGTGTAC	In vitro transcription of *lmo0514* wild type for EMSA assay
EMSA-0514-WT-R	AGTGGTTAATTTCTTCCCAAAGT	In vitro transcription of *lmo0514* wild type for EMSA assay
EMSAT7-5´sbrA-F	TAATACGACTCACTATAGGAGTACATCTAGATCCATACCCCTAAAC	In vitro transcription of SbrA wild type for EMSA assay
EMSA-5´sbrA-R	AAAAAGAGCAGCACCCGA	In vitro transcription of SbrA wild type for EMSA assay

* Restriction sites underlined, artificial terminator in italics and underlined. Mutations introduced in Rli27 (cloned in pP1 vector) or in 5’UTR of lmo0514 indicated in blue.
